# Circulating SARS-CoV-2 Spike IgG Antibody Levels and Avidity in Autoimmune, IBD and Transplant Cohorts: An Observational Study Following Multiple COVID-19 Vaccine Boosters

**DOI:** 10.3390/vaccines14080688

**Published:** 2026-08-11

**Authors:** Huijing Xue, Troy J. Kemp, Hayley North, Ligia A. Pinto

**Affiliations:** Vaccine, Immunity and Cancer Directorate, Frederick National Laboratory for Cancer Research, Frederick, MD 21701, USA; huijing.xue@nih.gov (H.X.); kemptj@mail.nih.gov (T.J.K.); north.hayley@gmail.com (H.N.)

**Keywords:** COVID-19, SARS-CoV-2, autoimmune, IBD, transplant, serology, avidity, mRNA vaccine

## Abstract

**Background**: Individuals with autoimmune disease, inflammatory bowel disease (IBD), and transplants face a higher risk of severe COVID-19 and breakthrough hospitalizations. It is essential to understand the magnitude and durability of vaccine-induced humoral immune response in these populations. **Methods**: We evaluated SARS-CoV-2 spike IgG antibody levels and avidity in autoimmune, IBD, transplant, and healthy cohorts following multiple COVID-19 mRNA vaccine doses. Serum samples were collected approximately 1 month (8–52 days) and 6 months (158–202 days) post-vaccination. Antibody levels and avidity were measured using validated ELISA and chaotropic-based avidity assays. **Results**: Individuals with IBD and individuals with autoimmune disease, especially systemic autoimmune disease, exhibited lower antibody levels and avidity compared with healthy individuals at certain doses and time points. Transplant recipients demonstrated substantial impairments in both antibody levels and avidity, with avidity reduced across all doses and time points. Significantly lower avidity levels were observed in transplant recipients, suggesting challenges in developing or maintaining antibody quality. For example, geometric mean anti-spike IgG levels were substantially lower in transplant recipients than in healthy individuals at both 1 month (635 vs. 7685 BAU/mL; *p* < 0.0001) and 6 months (1146 vs. 3277 BAU/mL; *p* = 0.0057) post third dose. Similarly, transplant recipients had lower antibody avidity than healthy individuals after the third dose, with geometric mean AI80 values of 4.5 M vs. 5.5 M at 1 month (*p* < 0.0001) and 4.6 M vs. 5.4 M at 6 months (*p* < 0.0001). Age, sex, and vaccine manufacturer may further influence humoral immune responses in the transplant cohort. **Conclusions**: Vaccine-induced humoral immunity varies across autoimmune, IBD, and transplant cohorts, with the most persistent impairment observed in transplant recipients across vaccine dose groups and at both post-vaccination time points. These findings reveal immune response patterns that may guide future studies evaluating vaccination schedules, immune monitoring, and clinical outcomes in these populations.

## 1. Introduction

The rapid development and deployment of SARS-CoV-2 vaccines have significantly reduced the burden of COVID-19, particularly in reducing severe illness and mortality [1,2]. A key component of the protective efficacy of these vaccines is the generation of robust humoral immune responses, including the production of immunoglobulin G (IgG) antibodies targeting the spike (S) protein of the virus. However, critical gaps persist in understanding vaccine efficacy among immunocompromised populations, which were excluded from pivotal clinical trials [3].

In the United States, over 15 million people live with at least one autoimmune disease [4]. An estimated 2.4 to 3.1 million Americans have been diagnosed with inflammatory bowel disease (IBD) [5], and over 48,000 transplants were performed nationwide in 2024 [6]. These individuals face high risks of severe COVID-19 outcomes, driven by intrinsic immune dysregulation or immunosuppressive treatments that can impair protective responses against infection and disease [7,8,9]. Furthermore, immunocompromised individuals account for nearly half of the breakthrough hospitalizations post-vaccination [10]. Despite their vulnerability, comparative analyses of vaccine-induced immunity in these populations remain strikingly limited, perpetuating knowledge gaps that may underestimate their need for tailored interventions.

Emerging data highlight that individuals with autoimmune diseases such as rheumatoid arthritis or multiple sclerosis often exhibit suboptimal immune responses to both the primary vaccination series and booster doses [11,12,13]. Similarly, IBD patients undergoing active therapy show impaired humoral immune response after boosters [14]. Transplant recipients, who typically require lifelong immunosuppression, also exhibited diminished antibody responses [15,16]. While previous research compared the seroconversion or antibody levels in individual cohorts with specific vaccine doses, a standardized understanding of the magnitude and durability of antibody response in different cohorts is limited, particularly after multiple booster doses. In addition, data on antibody quality measures such as avidity, which are important indicators of immune protection, are lacking.

In this study, we evaluated circulating SARS-CoV-2 spike-specific IgG antibody levels and antibody avidity in individuals with autoimmune disease, inflammatory bowel disease, and transplant recipients following two to five COVID-19 mRNA vaccine doses. Anti-spike IgG levels were measured using a validated and standardized assay according to the Clinical and Laboratory Standards Institute (CLSI) guidelines [17], and reported in binding antibody units per milliliter (BAU/mL), calibrated to the WHO International Standard [18], to support harmonized reporting and facilitate comparisons with other studies. In parallel, antibody avidity was assessed to provide additional information on antibody quality and maturation following repeated vaccination. By measuring antibody levels and avidity at 1- and 6-month intervals following each vaccine dose (from the second through the fifth dose), our study provides detailed insight into vaccine immunogenicity and durability, enabled by harmonized sampling protocols and centralized immune profiling through participating Serological Sciences Network (SeroNet) hospitals and clinics.

## 2. Materials and Methods

### 2.1. Study Design and Participants

This observational serological study followed the general study design established in our previous work [19] but evaluated distinct clinical populations. In brief, the study included serum samples from participants with autoimmune diseases (*N* = 81), IBD (*N* = 44), or a history of transplantation (*N* = 113). Three SeroNet Capacity Building Centers provided these samples: the University of Minnesota under Medical Protocol HRP-590, the Icahn School of Medicine at Mount Sinai under Protocol STUDY-16-01215, and the Feinstein Institutes for Medical Research under Institutional Review Board protocol #20-1007. The healthy comparison cohort comprised 428 donors recruited through these three centers and through Occupational Health Services at the Frederick National Laboratory for Cancer Research in Frederick, Maryland, under Research Donor Protocol OH99CN046. Participants were enrolled from January 2021 through January 2023, and serum specimens were obtained between January 2021 and August 2024. Across all cohorts, participants were eligible for inclusion if they were at least 18 years old, able to provide informed consent, had received at least two doses of a SARS-CoV-2 vaccine, and had no evidence of prior SARS-CoV-2 infection based on available clinical history. Individuals were excluded if they had evidence of prior SARS-CoV-2 infection. Medication-related exclusion criteria and minimum time post-transplant criteria were not applied. Participants were grouped according to their clinical cohort and the most recent vaccine dose received. For each vaccine dose group, samples were collected separately from participants whose most recent vaccination corresponded to that dose number. Blood samples were taken approximately 1 month (8–52 days) and 6 months (158–202 days) after the latest vaccination; however, no transplant cohort samples were available for two doses during any time points. Due to enrollment timing constraints, detailed vaccination histories beyond the number of doses received were generally unavailable for most subjects; therefore, analyses regarding vaccine manufacturers relied on the last administered dose. This was an observational serological study rather than a fully longitudinal repeated-measures study following the same participants sequentially from the second through fifth vaccine doses. Therefore, comparisons across vaccine dose groups should be interpreted as cross-sectional comparisons of participants sampled after different most recent vaccine doses, rather than as within-individual longitudinal changes across all vaccine doses.

### 2.2. Sample Preparation

Serum preparation followed the previously established procedure [19]. Whole blood was drawn into serum separator tubes and maintained upright at room temperature for 30–60 min to permit clot formation. The tubes were subsequently centrifuged at 1300× *g* for 20 min at room temperature. The serum fraction was transferred into sterile cryovials and placed at −80 °C. All samples were processed and frozen on the day of collection. Before serological testing, frozen serum was thawed, aliquoted, and incubated at 56 °C for 30–60 min for heat inactivation.

### 2.3. Enzyme-Linked Immunosorbent Assays (ELISA)

Serum IgG directed against the SARS-CoV-2 spike protein was measured using the standardized ELISA platform previously described [19]. The assay antigen was the full-length spike protein derived from the ancestral Wuhan SARS-CoV-2 strain.

For plate preparation, 100 μL of recombinant SARS-CoV-2 spike protein at 0.3 μg/mL in phosphate-buffered saline (PBS) was dispensed into each well of Maxisorp 96-well plates (Thermo Scientific, Waltham, MA, USA, Cat# 439454). The recombinant antigen was produced by the Protein Expression Laboratory at the Frederick National Laboratory for Cancer Research. Plates were incubated at 4 °C for at least 24 h. This coating concentration was selected based on internal assay optimization and lot-specific performance evaluation of spike proteins used in our laboratory, as described in our previous publication [17]. Following coating, plates were washed three times with PBS/Tween buffer. To reduce nonspecific binding, wells were blocked for 90 min with PBS/Tween-based blocking buffer supplemented with 4% skim milk. Heat-inactivated serum samples were diluted in blocking buffer beginning at 1:150 or higher, followed by threefold serial dilutions performed in the assay plate. Diluted samples were incubated on the coated plates for 60 min at room temperature. Spike-specific IgG binding was detected using goat anti-human IgG HRP-conjugate (Seracare, Milford, MA, USA, Cat# 5220-0390), followed by development with tetramethylbenzidine (TMB) substrate (Seracare, Milford, MA, USA, Cat# 5120-0049, 5120-0038). Color development was terminated by adding 0.36 N sulfuric acid. The signal was measured at 450 nm and 620 nm with a SpectraMax plate reader (Molecular Devices, San Jose, CA, USA). The resulting data were processed with SoftMax Pro GxP 7.0.3. Quantitative results were reported as binding antibody units per milliliter (BAU/mL), calibrated to the World Health Organization (WHO) International Standard (IS) [17,18].

### 2.4. Avidity Enzyme-Linked Immunosorbent Assays (Chaotrope ELISA)

Spike-specific IgG avidity was assessed using a chaotrope-based ELISA adapted from the quantitative IgG assay described above and previously reported [19]. This assay incorporates a chaotrope-disruption step after antibody binding to distinguish more weakly bound antibodies from antibodies with stronger antigen-binding interactions. Urea was selected as the chaotropic reagent because it provides an appropriate disruption range while preserving the integrity of the spike protein coating on the assay plate. For avidity testing, heat-inactivated serum samples were diluted to generate optical density (OD) values within the assay’s acceptable working range of 0.5 to 1.3 at 450 nm, with an approximate target OD of 1.0. After diluted samples were incubated on spike-coated plates, the plates were washed and then treated with increasing concentrations of urea, ranging from 0 to 10 M, to disrupt lower-avidity antibody–antigen interactions. Following urea treatment, plates were washed again and processed using the same detection and development steps as described for the quantitative IgG ELISA. Avidity indices (AI80), defined as the molar concentration of urea required to reduce the signal to 80% of untreated wells, were reported. Low- and high-avidity control sera ensured proper performance throughout each assay run. Prior assay development using multiple SARS-CoV-2 serum samples demonstrated reproducible avidity measurements with coefficients of variation below 10%, as previously reported [20].

### 2.5. Data Analysis

Statistical analyses were performed to compare SARS-CoV-2 spike-specific IgG levels and antibody avidity between healthy participants and participants in the autoimmune disease, IBD, and transplant cohorts. Because the data were not normally distributed, pairwise comparisons between each clinical cohort and healthy controls were performed using Wilcoxon rank-sum tests within each vaccine dose and post-vaccination interval. For the primary cohort comparisons, *p* values were adjusted using the Benjamini–Hochberg false discovery rate method within each vaccine dose and post-vaccination interval. Statistical analyses were performed in R version 4.3.1, with *p* values below 0.05 considered statistically significant. Anti-spike IgG levels and avidity were reported as geometric means (GMs) with corresponding 95% confidence intervals (CIs). The proportion of participants positive for anti-nucleocapsid antibodies was compared between cohorts using Pearson’s Chi-squared test. Changes in antibody responses between 1 month and 6 months post-vaccination were assessed using the percent change calculated for each individual at a given dose. Percent changes were reported as the median with the first and third quartiles (Q1, Q3). Exploratory subgroup analyses by disease subgroup, age, sex, transplant type, and most recent vaccine manufacturer were interpreted descriptively due to modest and uneven subgroup sizes. Comparisons were limited to within-cohort assessments. Formal interaction testing and regression-based adjustment were not performed.

## 3. Results

### 3.1. Demographic Characteristics of the Study Participants

Anti-SARS-CoV-2 spike IgG levels and avidity were measured in serum samples from healthy participants (*N* = 428) and participants in the autoimmune disease (*N* = 81), IBD (*N* = 44), and transplant cohorts (*N* = 113, including both solid organ and hematopoietic stem cell transplant recipients). Participant demographic characteristics, including age, sex, race, and ethnicity, are summarized in Table 1, with treatment details provided in Supplementary Table S1. Because treatment information was incomplete for many participants, available treatment data are presented only descriptively. The cohorts received two to five vaccine doses, including the primary series and up to three boosters.

### 3.2. Anti-Spike IgG Level After Vaccination in Autoimmune, IBD, and Transplant Cohorts

**Figure 1:**
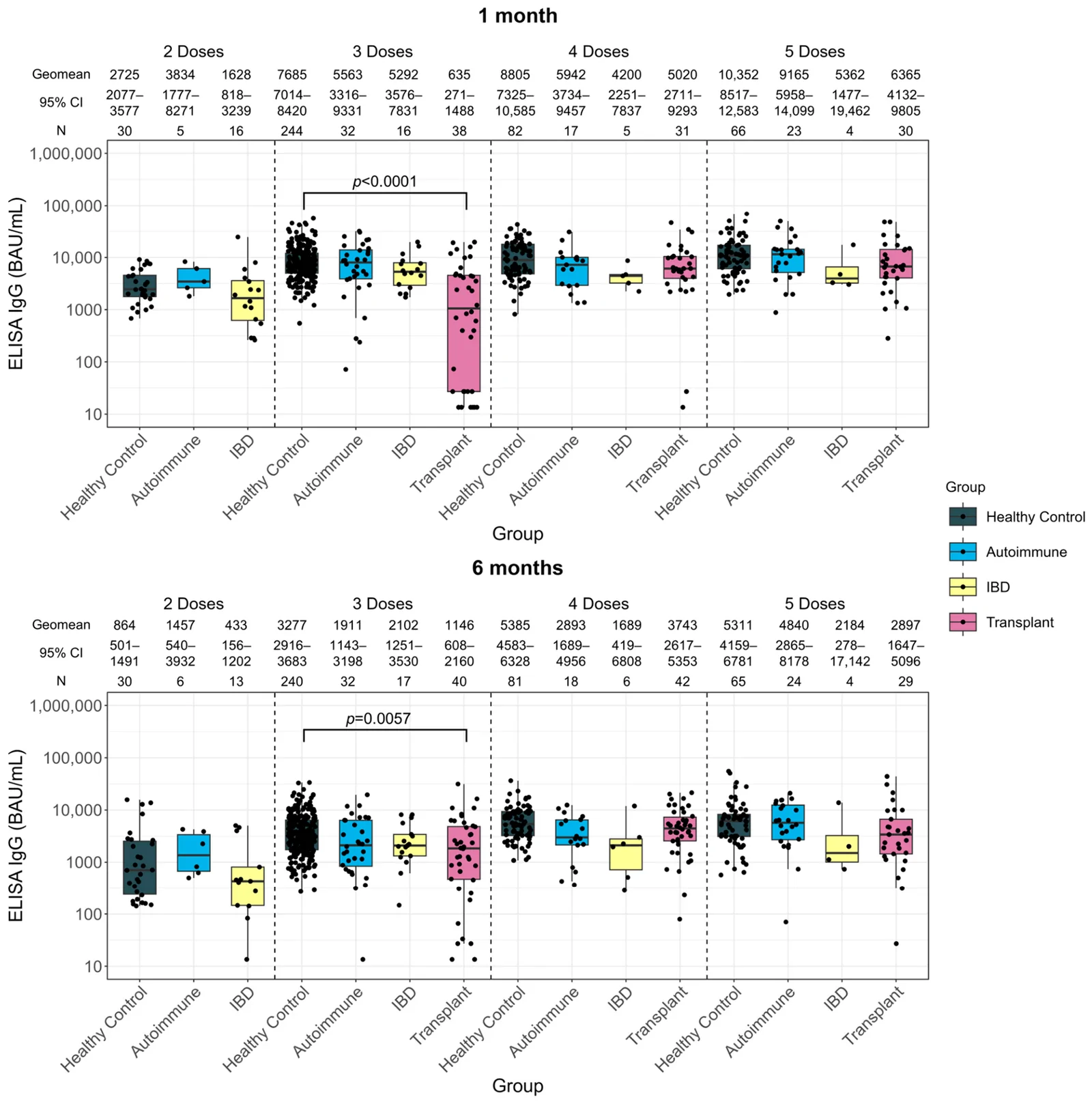
Serum anti–SARS-CoV-2 spike IgG responses following vaccination in the autoimmune disease, IBD, transplant, and healthy cohorts. Anti-spike IgG levels were measured in serum samples from the healthy, autoimmune, IBD, and transplant cohorts at 1 month and 6 months post-vaccination. Data are summarized as GM with 95% CI. Box plots depict the median (center line) and interquartile range. Group comparisons were performed using the non-parametric Wilcoxon rank-sum test. *p* values were adjusted using the Benjamini–Hochberg false discovery rate method within each dose and post-vaccination interval. Adjusted *p* values < 0.05 were considered statistically significant.

In the autoimmune cohort (Figure 1, Supplementary Table S2), no significant differences in spike IgG levels were observed relative to healthy individuals across different doses at 1 month and 6 months post-vaccination. Because systemic autoimmune diseases may involve broader immune dysregulation and different treatment patterns than non-systemic autoimmune conditions, the autoimmune cohort was further subgrouped into systemic autoimmune disease and non-systemic autoimmune disease cohorts (Supplementary Figure S1). Specifically, the systemic autoimmune disease group included participants with systemic lupus erythematosus, rheumatoid arthritis, and Sjögren syndrome. The non-systemic autoimmune diseases were restricted to a single organ or tissue (e.g., arthropathic psoriasis, Hashimoto’s disease, vitiligo) (Supplementary Table S3). Significantly lower anti-spike IgG levels were noted in the systemic autoimmune cohort at 1 month post fourth dose (2859 BAU/mL, *p* = 0.0142) and 6 months post third dose (755 BAU/mL, *p* = 0.0093) compared to healthy individuals. In contrast, no significant differences were observed in the non-systemic autoimmune cohort.

The IBD cohort (Figure 1, Supplementary Table S2) showed no significant differences in spike IgG levels compared with healthy individuals across vaccine doses at 1 month and 6 months post-vaccination.

The transplant cohort (Figure 1, Supplementary Table S2) had significantly lower anti-spike IgG responses than the healthy cohort post third dose at 1 month (635 vs. 7685 BAU/mL, *p* < 0.0001) and 6 months (1146 vs. 3277 BAU/mL, *p* = 0.0057). No significant differences were observed post fourth and fifth doses.

The transplant cohort was further subgrouped based on the transplant type (Supplementary Figure S2). Lower anti-spike IgG response was observed in kidney transplant patients post third dose (1 month: 600 BAU/mL, *p* < 0.0001; 6 months: 578 BAU/mL, *p* = 0.0025) compared to healthy individuals. Liver transplant patients experienced lower antibody levels at 1 month post third dose (792 BAU/mL, *p* = 0.0013) than healthy individuals. Lower anti-spike IgG levels were also observed in heart transplant recipients despite small sample sizes.

In addition to comparisons with the overall healthy group, age- and sex-matched healthy-control comparisons were performed for each clinical cohort where feasible, and the corresponding *p* values are reported in Supplementary Table S2. However, because matching reduced the available sample size and statistical power, these analyses were considered supportive.

Anti-nucleocapsid IgG antibody results indicated no significant differences between healthy participants and participants in the autoimmune disease, IBD, and transplant cohorts (Supplementary Table S4), suggesting that previous detectable SARS-CoV-2 infection or breakthrough infection did not substantially influence the observed vaccine-induced antibody responses.

### 3.3. Anti-Spike IgG Avidity After Vaccination in Autoimmune, IBD, and Transplant Cohorts

**Figure 2:**
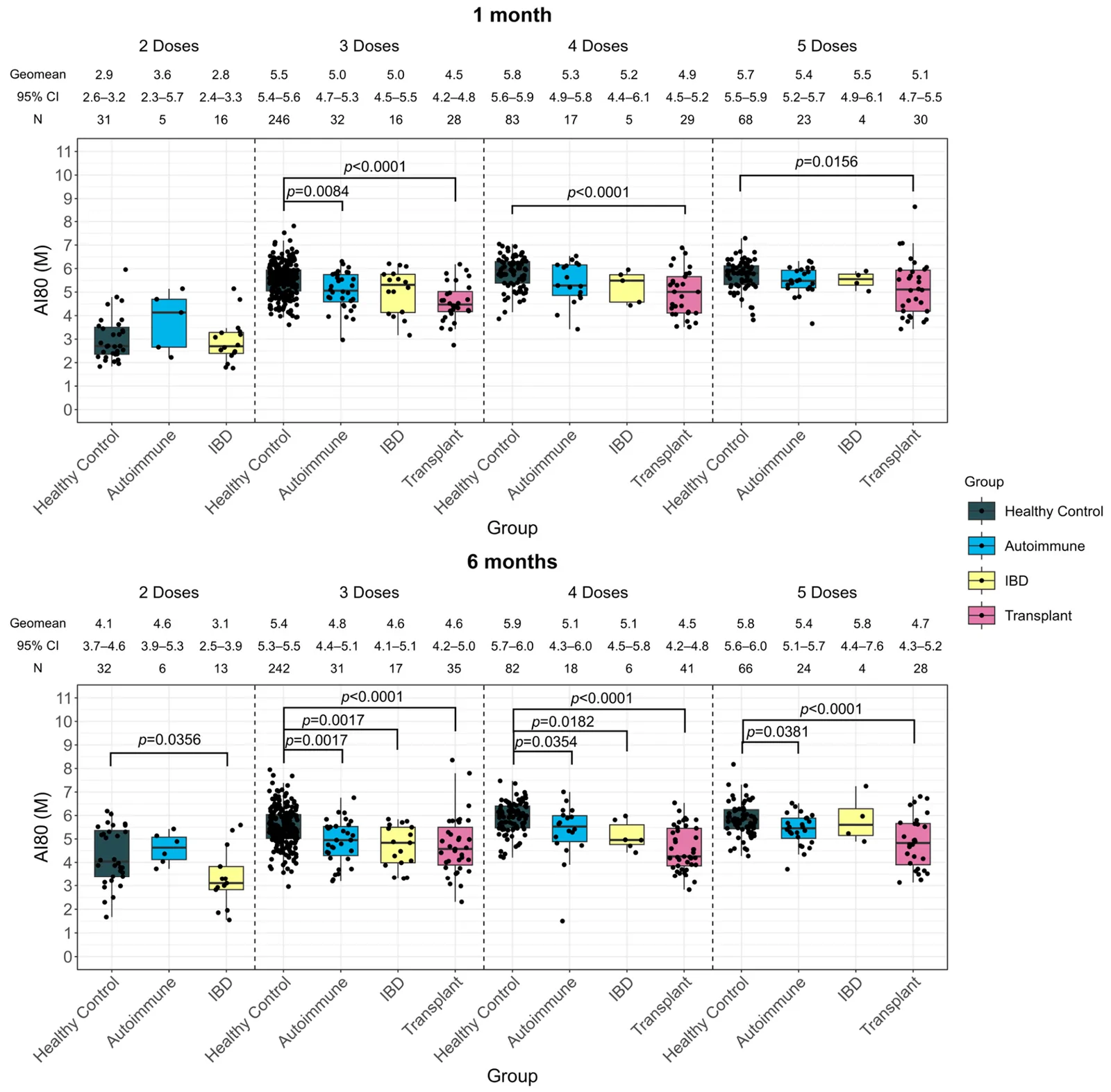
Serum anti–SARS-CoV-2 spike IgG avidity following vaccination in the autoimmune disease, IBD, transplant, and healthy cohorts. Anti-spike IgG avidity was evaluated in healthy controls and participants with autoimmune disease, IBD, and transplants at 1 and 6 months following vaccination. Data are summarized as GM with 95% CI. Box plots depict the median (center line) and interquartile range. Group comparisons were performed using the non-parametric Wilcoxon rank-sum test. *p* values were adjusted using the Benjamini–Hochberg false discovery rate method within each dose and post-vaccination interval. Adjusted *p* values < 0.05 were considered statistically significant.

At 1 month post third dose (Figure 2, Supplementary Table S5), avidity in the autoimmune cohort (5.0 M, *p* = 0.0084) was lower than in the healthy cohort (5.5 M). At 6 months, significantly reduced avidity was observed post third (4.8 M, *p* = 0.0017), fourth (5.1 M, *p* = 0.0354), and fifth (5.4 M, *p* = 0.0381) doses in the autoimmune participants compared to healthy individuals (third: 5.4 M; fourth: 5.9 M; fifth: 5.8 M). Subgroup analysis (Supplementary Figure S3) revealed that individuals with systemic autoimmune conditions exhibited lower avidity at both the 1- and 6-month time points post third dose, while non-systemic autoimmune participants showed reductions only at 6 months post third dose. No additional significant differences were observed.

Among the participants with IBD, avidity remained comparable to healthy individuals at 1 month post-vaccination but was significantly lower at 6 months post second (3.1 M, *p* = 0.0356), third (4.6 M, *p* = 0.0017), and fourth (5.1 M, *p* = 0.0182) doses (Figure 2, Supplementary Table S5).

The transplant cohort (Figure 2, Supplementary Table S5) consistently exhibited significantly lower avidity across all doses and time points relative to the healthy participants. At 1 month post-vaccination, the avidity of the transplant cohort was 4.5 M (*p* < 0.0001) post third dose, 4.9 M (*p* < 0.0001) post fourth dose, and 5.1 M (*p* = 0.0156) post fifth dose, vs. 5.5 M, 5.8 M, and 5.7 M in the healthy participants, respectively. At 6 months post-vaccination, avidity remained reduced in the transplant cohort (third: 4.6 M, *p* < 0.0001; fourth: 4.5 M, *p* < 0.0001; fifth: 4.7 M, *p* < 0.0001), while healthy individuals retained higher avidity (third: 5.4 M; fourth: 5.9 M; fifth: 5.8 M). Subgroup analyses by transplant type (Supplementary Figure S4) indicated that the kidney and liver recipients showed significantly diminished avidity at multiple doses and time points. Lung, heart, and multi-organ transplant recipients also exhibited impaired avidity at certain doses and time points, though sample sizes were limited.

Consistent with the anti-spike IgG analysis, age- and sex-matched healthy-control comparisons were also performed for antibody avidity where feasible, with corresponding *p* values reported in Supplementary Table S5.

### 3.4. Anti-Spike IgG Level Decay Rate and Avidity Dynamics in Autoimmune, IBD and Transplant Cohorts

**Figure 3:**
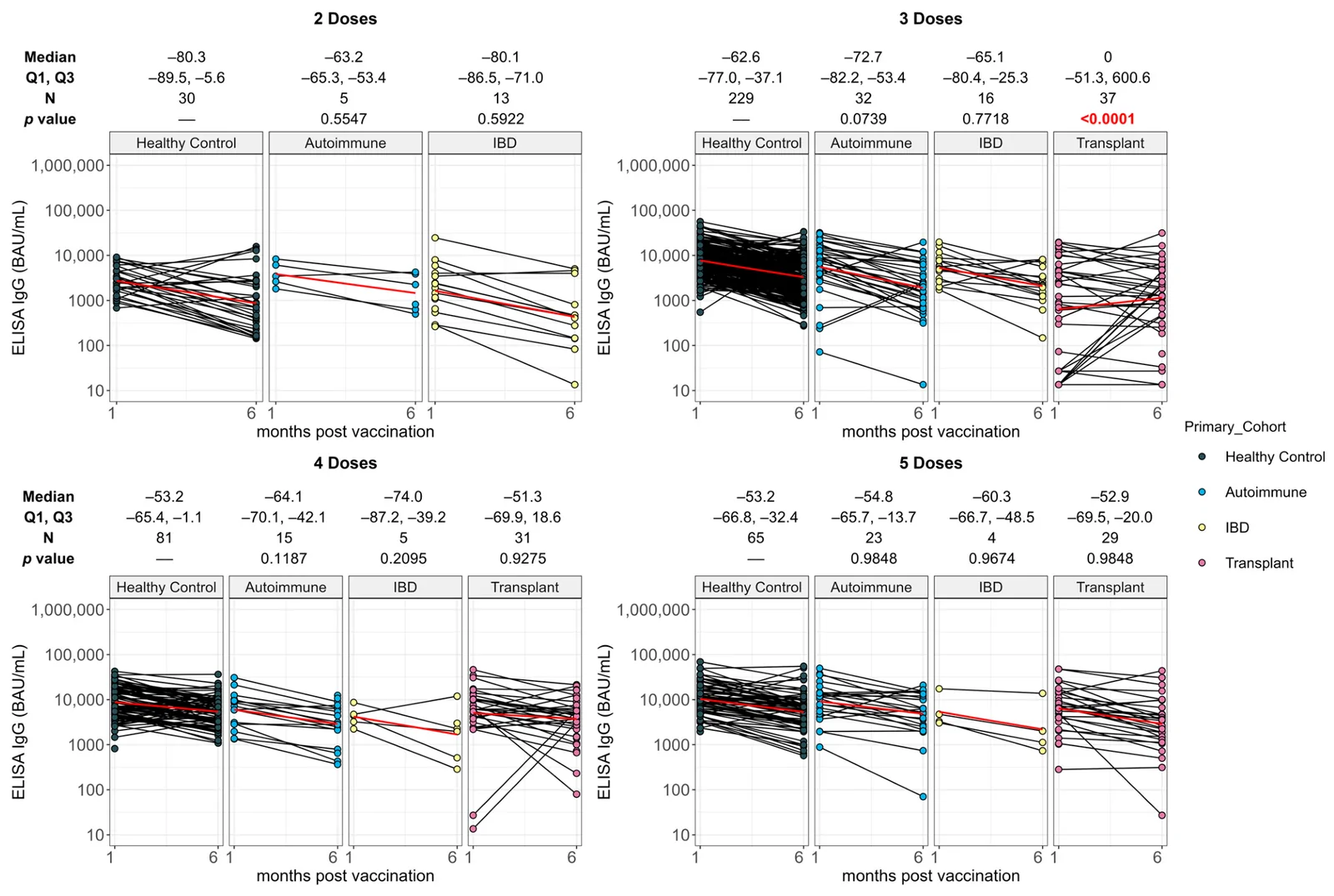
Percent change in anti-spike IgG levels between 1 month and 6 months following vaccination in the autoimmune disease, IBD, transplant, and healthy cohorts. The percent change in serum anti-spike IgG levels between 1 month and 6 months post-vaccination was assessed in healthy, autoimmune, IBD, and transplant participants who received 2, 3, 4, and 5 vaccine doses. Percent change results were summarized as median with Q1 and Q3. *p* values reflect comparisons of anti-spike IgG percent change in each patient cohort relative to the corresponding healthy cohort at matched vaccine doses. Solid red lines represent the trajectory connecting geometric mean IgG levels at 1 month and 6 months for each cohort. Group comparisons were performed using the non-parametric Wilcoxon rank-sum test. *p* values were adjusted using the Benjamini–Hochberg false discovery rate method within each dose. Adjusted *p* values < 0.05 were considered statistically significant and are indicated by bold red text.

To better understand the change in antibody levels and avidity between 1 month and 6 months post-vaccination, the percent change was calculated and compared between healthy participants and participants in the autoimmune disease, IBD, and transplant cohorts (Figure 3). No differences in the anti-spike IgG percent change between the autoimmune disease or IBD cohorts and the healthy cohort were noted. A significantly higher percent change in anti-spike IgG levels was observed in the transplant cohort post third dose (0%, *p* < 0.0001) compared to the healthy cohort (−62.6%). Among individuals with higher anti-spike IgG levels at 6 months than at 1 month after vaccination, we did not identify a consistent pattern of anti-nucleocapsid IgG positivity that explained the observed increase, although clinical breakthrough infection history was not available for confirmation.

**Figure 4:**
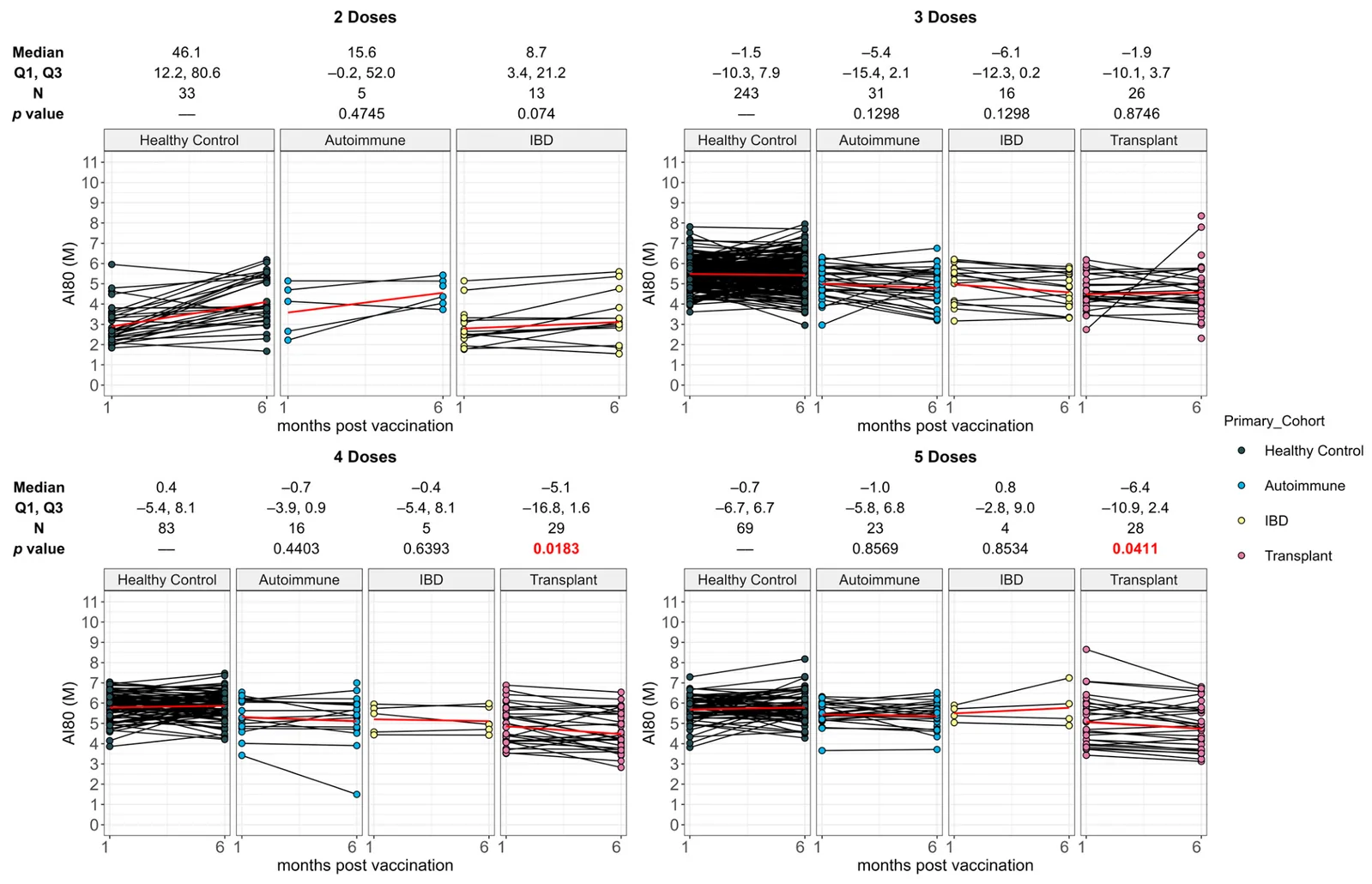
Percent change in anti-spike IgG avidity between 1 month and 6 months following vaccination in the autoimmune disease, IBD, transplant, and healthy cohorts. The percent change in serum anti-spike IgG avidity between 1 month and 6 months post-vaccination was assessed in healthy, autoimmune, IBD, and transplant participants who received 2, 3, 4, and 5 vaccine doses. Percent change results were summarized as median with Q1 and Q3. *p* values reflect comparisons of avidity percent change in each patient cohort relative to the corresponding healthy cohort at matched vaccine doses. Solid red lines represent the trajectory connecting geometric mean avidity at 1 month and 6 months for each cohort. Group comparisons were performed using the non-parametric Wilcoxon rank-sum test. *p* values were adjusted using the Benjamini–Hochberg false discovery rate method within each dose. Adjusted *p* values < 0.05 were considered statistically significant and are indicated by bold red text.

For the percent change in avidity between 1 month and 6 months (Figure 4), no significant differences were observed in the autoimmune and IBD cohorts. The transplant cohort exhibited significantly lower percent changes post the fourth (−5.1%, *p* = 0.0183) and fifth (−6.4%, *p* = 0.0411) doses when compared to the healthy cohort (fourth: 0.4%; fifth: −0.7%).

### 3.5. Exploratory Subgroup Analyses

#### 3.5.1. Influence of Age

To explore whether age, sex, and vaccine manufacturer contributed to differences among the clinical cohorts, participants were stratified by age (<65 vs. ≥65 years), sex (male vs. female), and manufacturer of the most recently received vaccine dose (Moderna mRNA-1273 vs. Pfizer-BioNTech BNT162b2). Anti-spike IgG levels and antibody avidity were then compared across the healthy, autoimmune disease, IBD, and transplant cohorts within each subgroup. Because these analyses were exploratory and subgroup sizes were modest, the results were interpreted descriptively.

**Figure 5:**
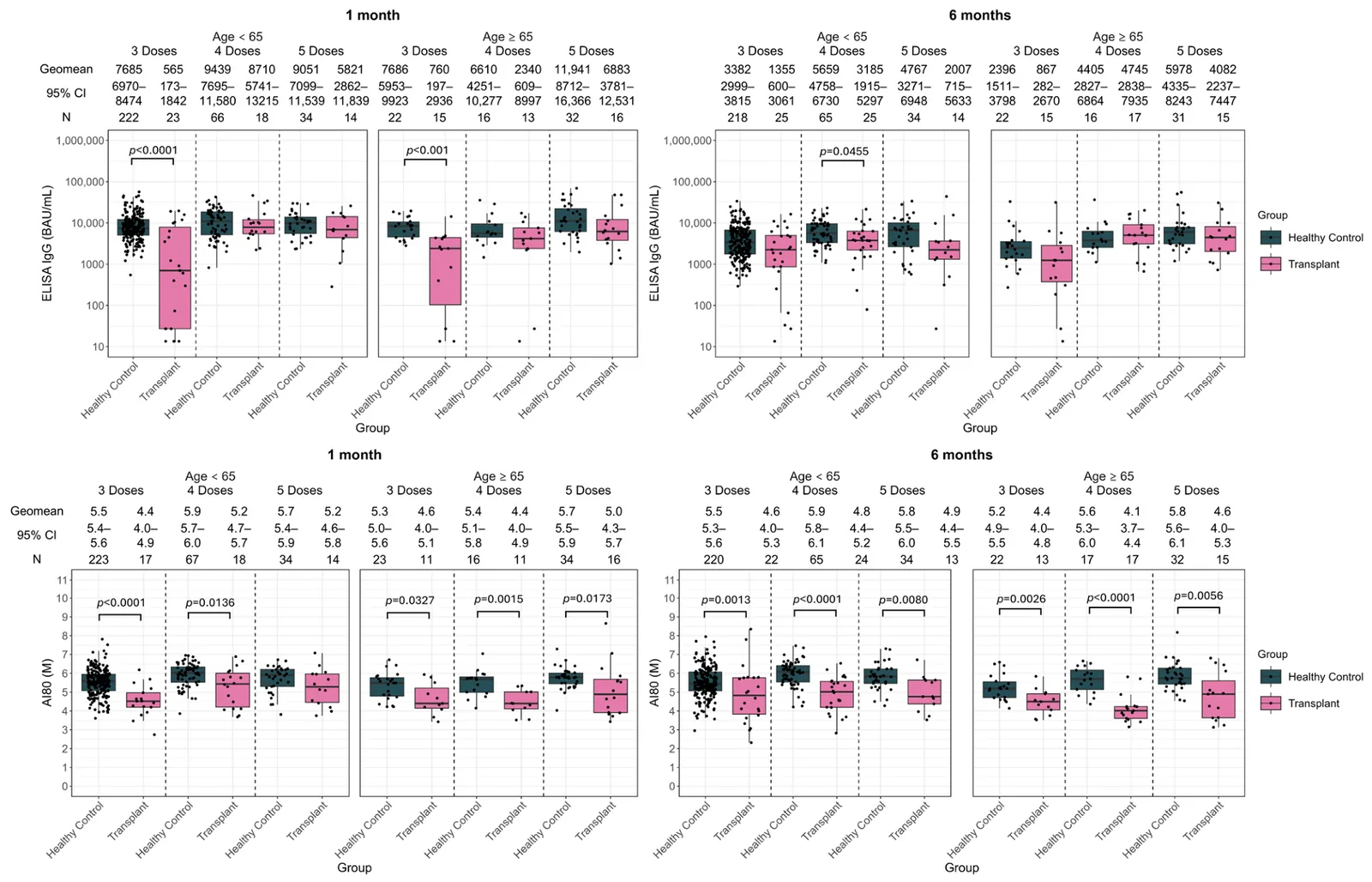
Age-stratified anti–SARS-CoV-2 spike IgG levels and antibody avidity. Serum anti-spike IgG levels (**upper panel**) and avidity (**lower panel**) were evaluated in samples from the healthy and transplant cohorts, stratified by age (<65 and ≥65 years). Data are summarized as GM with 95% CI. Box plots depict the median (center line) and interquartile range. Group comparisons within each age group for each cohort were performed using the non-parametric Wilcoxon rank-sum test.

Both younger (<65 years) and older (≥65 years) groups in the transplant cohort (Figure 5) exhibited lower anti-spike IgG levels at 1 month post third dose (younger: 565 BAU/mL, *p* < 0.0001; older: 760 BAU/mL, *p* < 0.001) when compared to the healthy cohort (younger: 7685 BAU/mL; older: 7686 BAU/mL). At 6 months post-vaccination, only the younger transplant cohort showed lower anti-spike IgG response post fourth dose (3185 BAU/mL, *p* = 0.0455) than the healthy cohort (5659 BAU/mL), while this trend was not observed in the older transplant cohort.

The transplant cohort in both age groups consistently exhibited lower avidity than healthy individuals across the third, fourth, and fifth doses at both the 1-month and 6-month time points (Figure 5). The only exception was the younger transplant cohort at 1 month post fifth dose, where no significant difference in avidity was noted compared to healthy individuals.

Further stratification by age in the autoimmune and IBD cohorts resulted in very small subgroup sizes, and the descriptive results are provided in Supplementary Figures S5 and S6.

#### 3.5.2. Influence of Sex

**Figure 6:**
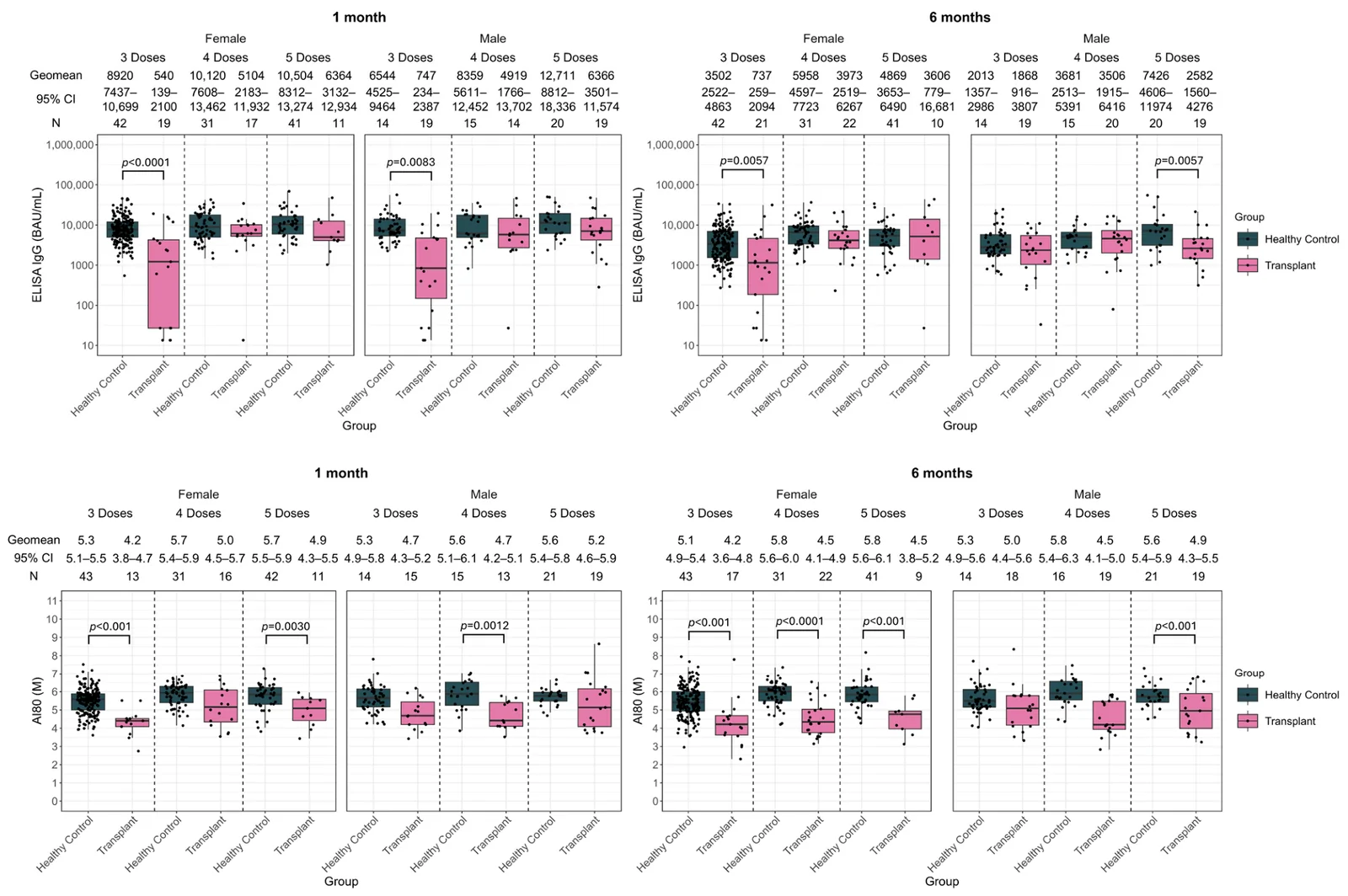
Sex-stratified anti–SARS-CoV-2 spike IgG levels and antibody avidity. Serum anti-spike IgG levels (**upper panel**) and avidity (**lower panel**) were evaluated in samples from the healthy and transplant cohorts, stratified by sex. Data are summarized as GM with 95% CI. Box plots depict the median (center line) and interquartile range. Group comparisons within each sex group for each cohort were performed using the non-parametric Wilcoxon rank-sum test.

Within the transplant cohort, female participants exhibited reduced anti-spike IgG levels compared with healthy controls at 1 month (540 BAU/mL vs. 8920 BAU/mL, *p* < 0.0001) and 6 months (737 BAU/mL vs. 3502 BAU/mL, *p* = 0.0057) post third dose (Figure 6). Male transplant recipients also had lower anti-spike IgG levels at 1 month post third dose (747 BAU/mL, *p* = 0.0083) and 6 months post fifth dose (2582 BAU/mL, *p* = 0.0057).

Analysis of antibody avidity (Figure 6) revealed that the female transplant cohort consistently demonstrated lower avidity than healthy individuals across all doses and all time points, with the exception of 1 month post fourth dose. Among males, lower avidity was noted at both 1 month and 6 months post fourth dose.

Due to very small subgroup sizes after sex stratification in the autoimmune and IBD cohorts, only descriptive results are presented in Supplementary Figures S7 and S8.

#### 3.5.3. Influence of Vaccine Manufacturer

**Figure 7:**
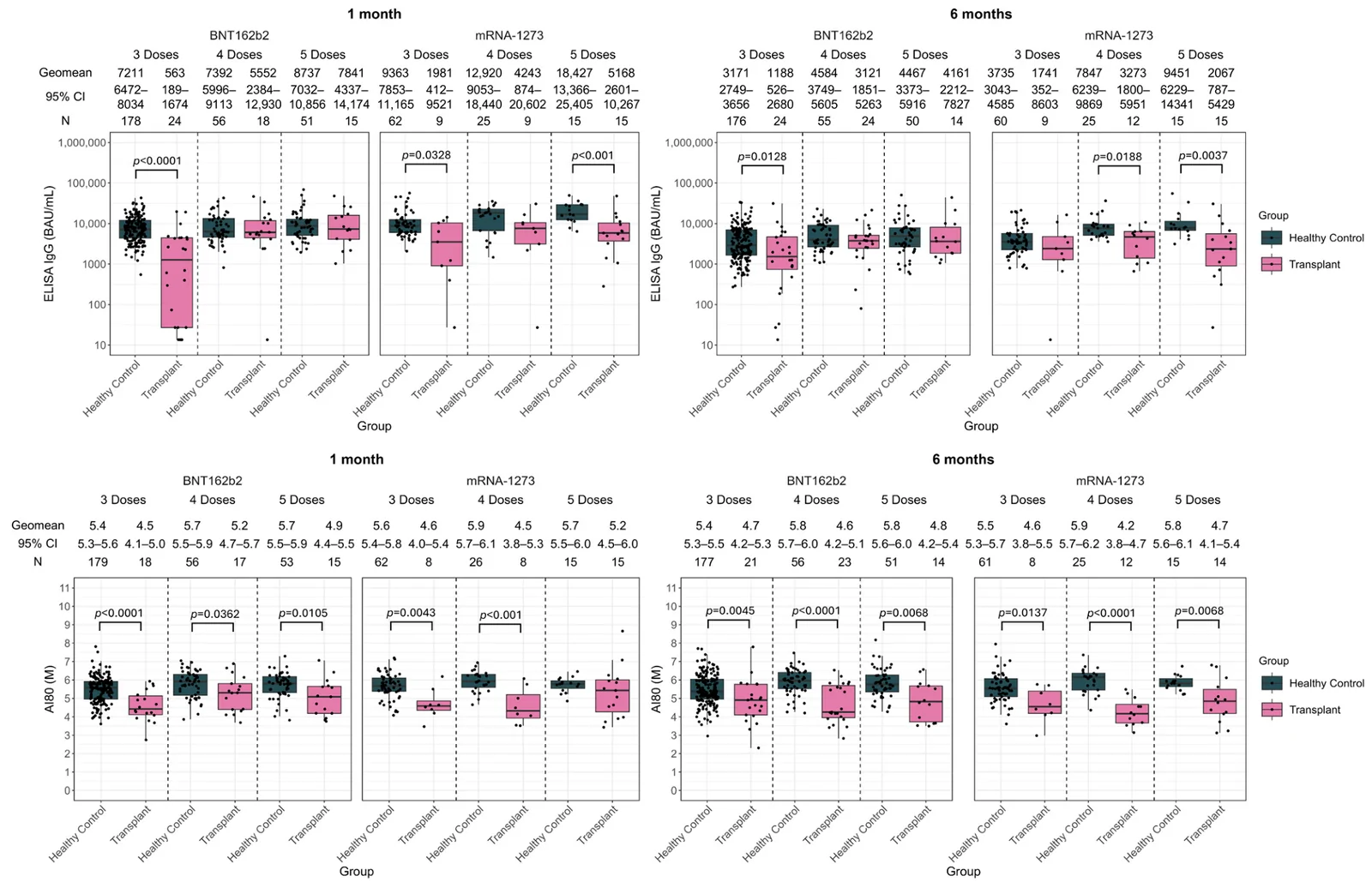
Vaccine-stratified anti–SARS-CoV-2 spike IgG levels and antibody avidity. Serum anti-spike IgG levels (**upper panel**) and avidity (**lower panel**) were evaluated in samples from the healthy and transplant cohorts, stratified by vaccine manufacturer. Data are summarized as GM with 95% CI. Box plots depict the median (center line) and interquartile range. Group comparisons within each vaccine group for each cohort were performed using the non-parametric Wilcoxon rank-sum test.

The BNT162b2 transplant cohort showed a lower antibody response at both 1 month (563 BAU/mL, *p* < 0.0001) and 6 months (1188 BAU/mL, *p* = 0.0128) post third dose when compared to the BNT162b2 healthy cohort (1 month: 7211 BAU/mL; 6 months: 3171 BAU/mL) (Figure 7). The mRNA-1273 transplant cohort exhibited lower anti-spike IgG levels at 1 month post the third (1981 BAU/mL, *p* = 0.0328) and fifth (5168 BAU/mL, *p* < 0.001) doses compared to healthy individuals (third: 9363 BAU/mL; fifth: 18,427 BAU/mL). At 6 months post-vaccination, the mRNA-1273 vaccine recipient transplant cohort showed lower anti-spike IgG levels post the fourth (3273 BAU/mL, *p* = 0.0188) and fifth (2067 BAU/mL, *p* = 0.0037) doses when compared to healthy individuals (fourth: 7847 BAU/mL; fifth: 9451 BAU/mL).

The transplant cohort in both vaccine groups consistently exhibited lower avidity than healthy individuals across the third, fourth, and fifth doses at both the 1-month and 6-month time points, except for transplant recipients who received the mRNA-1273 vaccine at 1 month post fifth dose (Figure 7).

Further stratification by vaccine manufacturer in the autoimmune and IBD cohorts resulted in very small subgroup sizes; corresponding descriptive results are provided in Supplementary Figures S9 and S10.

## 4. Discussion

Despite the widespread availability of SARS-CoV-2 vaccines, individuals with autoimmune disease, inflammatory bowel disease (IBD), and transplant recipients remain at increased risk of breakthrough infections and severe COVID-19 outcomes. Serum IgG levels have been shown to play an important role in protection, with higher levels inversely correlated with infection risk and positively associated with vaccine efficacy [21,22,23]. Although vaccine-induced antibody responses decline within 3–6 months of vaccination, additional boosters can restore and enhance these responses [24,25,26]. In contrast, avidity typically increases with time, potentially due to the persistent activation of B-cell germinal centers that promotes the selection and survival of B cells, producing antibodies with stronger binding capacity to the target antigen [20,27]. Higher-avidity antibodies may exhibit broadened neutralizing capacity against SARS-CoV-2 variants of concern (VOCs) [28]. A protective humoral response depends on both the quantity and quality of circulating antibodies. However, standardized assessments integrating both antibody concentration and avidity across repeated booster doses in clinically distinct populations remain limited. In this context, our study adds to the existing literature by evaluating SARS-CoV-2 spike-specific IgG levels and antibody avidity from the second through fifth vaccine doses in autoimmune disease, IBD, and transplant cohorts compared with healthy individuals. Importantly, antibody concentrations were measured using a validated and standardized assay and reported in binding antibody units per milliliter (BAU/mL), calibrated to the WHO International Standard, which supports harmonized reporting and facilitates comparison across studies.

In this study, although the autoimmune cohort overall did not show significantly reduced anti-spike IgG levels compared with healthy individuals, antibody avidity remained impaired, suggesting that antibody maturation may be affected even when overall anti-spike IgG quantity is preserved. Subgroup analysis further suggests that reductions in anti-spike IgG levels may be more evident among participants with systemic autoimmune diseases. This difference may reflect clinical and immunological heterogeneity within the autoimmune cohort. Systemic autoimmune diseases often involve multi-organ immune dysregulation and may require stronger or combination immunosuppressive therapy, whereas non-systemic autoimmune diseases are more localized and often managed with less intensive treatment. These results align with previous studies showing reduced antibody levels and seroconversion rates in certain autoimmune disease populations [12,29,30]. However, treatment information was incomplete in the present cohort, preventing treatment-stratified analyses. Prior studies have shown that B-cell-depleting monoclonal antibodies such as rituximab are strongly associated with diminished antibody responses after the administration of mRNA vaccines [31,32]. Other therapies, including corticosteroids (prednisone ≥ 7.5 mg/day), mycophenolate mofetil (MMF), methotrexate, tumor necrosis factor (TNF) inhibitors, tocilizumab, and abatacept, have also been shown to reduce vaccine-induced antibody levels [32,33]. However, because treatment information was incomplete for the study cohorts, the influence of treatment on the observed antibody and avidity patterns could not be determined. Treatment timing plays an important role. Interval since the last rituximab infusion was the main factor influencing humoral response in the rituximab-treated autoimmune cohort [31]. Some studies suggest that methotrexate withdrawal for 2 weeks following COVID-19 vaccination improves the antibody levels, while it may increase the risk of disease flares [34,35,36]. Therefore, optimizing vaccination schedules in autoimmune cohorts requires the careful consideration of immunosuppressive therapy timing to maximize immune responses while minimizing clinical risks.

The IBD cohort in our study had significantly lower avidity at certain doses at 6 months, despite the small sample size. This suggests that antibody binding quality may be affected in some individuals with IBD. Diminished responses in the IBD cohort have been largely linked to anti-TNF therapies, which could be due to reduced or absent circulating T follicular helper 1 (cTfh1) activation and diminished B memory cell formation [37,38,39]. Combination therapy with thiopurine could further exacerbate this impairment [40]. For other therapies, previous studies have suggested attenuated responses with tofacitinib treatment and preserved responses with vedolizumab or ustekinumab treatment [37].

Among the evaluated cohorts, transplant recipients showed the most consistent impairment in both anti-spike IgG levels and antibody avidity. These findings align with previous studies showing that transplant recipients generally have lower seroconversion rates and reduced antibody titers following SARS-CoV-2 vaccination even with boosters [41,42]. Reduced avidity in transplant recipients may indicate impaired maturation or maintenance of antibody binding quality. When the transplant cohort was stratified by transplant type, some subgroup differences were observed, but these analyses were limited by small subgroup sizes and should be considered exploratory. In particular, the sample size of the hematopoietic stem cell transplant subgroup was very small, precluding comparisons with solid organ transplant recipients. Information on time since transplantation was also not consistently available. Besides the transplant type, variations in immunosuppressive treatments may also contribute to different trends across organ types, although treatment information was incomplete for the present cohort. Immunosuppressive treatments, including calcineurin inhibitors, MMF, belatacept, and corticosteroids, are associated with impaired antibody response by blunting B-cell activation and germinal center formation [43,44,45]. MMF appears to be particularly detrimental, with higher-intensity use associated with a dose-dependent reduction in antibody levels [46,47,48].

Older age and male sex are generally recognized as risk factors in the healthy population, and older age has also been associated with reduced immune responses in immune-altering conditions [37,44,49]. However, whether individuals in autoimmune disease, IBD, and transplant cohorts show additional impairment compared with healthy individuals of the same age or sex remains less well-understood. In this study, exploratory subgroup analyses suggest that differences between transplant recipients and healthy individuals may vary by age, sex, and most recent vaccine manufacturer. These analyses should be interpreted cautiously because subgroup sizes were modest, and complete vaccine manufacturer histories were unavailable. Although vaccine manufacturer analyses were based on the most recent dose and therefore may not reflect each participant’s complete vaccination history, these analyses still provide useful preliminary information regarding antibody responses after the latest vaccine exposure. These analyses may help identify potential contributors to heterogeneity in antibody quantity and avidity. Overall, these findings provide hypothesis-generating evidence and a rationale for future studies with larger sample sizes, complete vaccination histories, and regression-based interaction analyses to determine whether age, sex, or vaccine manufacturer modifies antibody responses in transplant recipients and other cohorts.

This study has several limitations. First, vaccination records were not available for earlier doses in many participants. Therefore, vaccine manufacturer analyses relied on the most recent dose received, and manufacturer-specific findings should be interpreted as exploratory and may not reflect the full vaccination history of each participant. Formulation-specific vaccination information, including whether individual doses were original monovalent or bivalent booster vaccines, was also not uniformly available across participants. Thus, we could not directly assess the impact of vaccine composition on anti-spike IgG levels or antibody avidity. Nevertheless, stratification by the most recent dose remains informative because it reflects antibody responses after repeated vaccine exposure and captures the cumulative boosting history of the participants. In addition, this was an observational serological study rather than a fully longitudinal repeated-measures study. Participants were grouped according to the most recent vaccine dose received, and comparisons across dose groups were primarily cross-sectional. Therefore, differences in participant characteristics, disease status, treatment exposure, vaccination history, and other unmeasured factors may have contributed to the observed differences across dose groups. Analyses in the autoimmune disease and IBD cohorts were limited by modest sample sizes at certain vaccine doses. Further stratification by age, sex, or vaccine type reduced the available sample size even more, limiting statistical power and preventing reliable regression-based interaction analyses.

Moreover, treatment type, dose, duration, and timing relative to vaccination were incompletely reported. Available treatment data were presented only descriptively, and relevant stratified analyses could not be performed, limiting evaluation of the potential impact of specific therapies or disease severity on vaccine-induced immune responses. Clinical breakthrough infection history was also not available. Finally, this study measured only anti-spike IgG levels and antibody avidity against the ancestral SARS-CoV-2 spike antigen. Therefore, our findings reflect the magnitude and binding quality of circulating spike-specific IgG responses, but they do not directly measure the functional neutralization activity, variant-specific antibody breadth or clinical protection. Cellular immunity is also an important component of vaccine-induced protection. Prior studies have shown that T-cell responses may be preserved despite suboptimal anti-spike IgG responses after booster vaccination, although this varies by treatment [50,51,52]. Transplant recipients generally show weaker vaccine-induced T-cell responses, particularly with immunosuppressive therapies such as mycophenolate, although spike-specific T-cell responses may still be detectable in some seronegative individuals [53,54,55]. Thus, while our study provides standardized evidence of anti-spike IgG levels and avidity, future studies incorporating neutralization assays, variant-specific antigen panels, cellular immune analyses, complete vaccination and formulation histories, documented infection history, as well as detailed treatment and disease severity information are needed to better define vaccine-induced immunity.

## 5. Conclusions

In summary, this study provides standardized serological evidence of SARS-CoV-2 spike-specific IgG responses and antibody avidity across up to five vaccine doses, offering detailed insight into vaccine-induced humoral responses in individuals with autoimmune diseases, IBD, and transplants. These cohorts exhibit varying degrees of impairment in anti-spike IgG levels and antibody avidity after multiple mRNA SARS-CoV-2 vaccine doses. Among the evaluated cohorts, transplant recipients showed the most consistent deficits in anti-spike IgG levels and antibody avidity. Exploratory analyses suggest that age, sex, and vaccine manufacturer may contribute to the differences observed between the healthy and transplant cohorts, whereas small sample sizes in the autoimmune and IBD cohorts limited the assessment of these factors. Collectively, these findings may guide future studies evaluating vaccination schedules and immune monitoring, and their relationship to durable protection in autoimmune disease, IBD, and transplant cohorts.

## Figures and Tables

**Table 1:** Demographic features of the study participants.

	Autoimmune (*N* = 81)	IBD (*N* = 44)	Transplant (*N* = 113)	Healthy (*N* = 428)	Total (*N* = 666)
**Age**					
Mean (SD)	51.5 (13.5)	44.2 (15.2)	60.0 (11.3)	48.7 (14.4)	50.6 (14.5)
Range	24–75	22–76	24–77	19–77	19–77
**Gender**					
Female	71 (87.7%)	35 (79.5%)	54 (47.8%)	311 (72.7%)	471 (70.7%)
Male	10 (12.3%)	9 (20.5%)	59 (52.2%)	117 (27.3%)	195 (29.3%)
**Race**					
American Indian or Alaska Native	0 (0.0%)	0 (0.0%)	1 (0.9%)	1 (0.2%)	2 (0.3%)
Asian	1 (1.2%)	2 (4.5%)	8 (7.1%)	52 (12.1%)	63 (9.5%)
Black or African American	9 (11.1%)	3 (6.8%)	7 (6.2%)	27 (6.3%)	46 (6.9%)
Multirace	2 (2.5%)	0 (0.0%)	1 (0.9%)	14 (3.3%)	17 (2.6%)
Other	1 (1.2%)	0 (0.0%)	11 (9.7%)	17 (4.0%)	29 (4.4%)
Unknown	1 (1.2%)	0 (0.0%)	0 (0.0%)	4 (0.9%)	5 (0.8%)
White	67 (82.7%)	39 (88.6%)	85 (75.2%)	313 (73.1%)	504 (75.7%)
**Ethnicity**					
Hispanic or Latino	7 (8.6%)	1 (2.3%)	13 (11.5%)	24 (5.6%)	45 (6.8%)
Not Hispanic or Latino	74 (91.4%)	26 (59.1%)	100 (88.5%)	379 (88.6%)	579 (86.9%)
Unknown	0 (0.0%)	17 (38.6%)	0 (0.0%)	25 (5.8%)	42 (6.3%)
**Dose**					
2	6 (7.4%)	16 (36.4%)	0 (0.0%)	30 (7.0%)	52 (7.8%)
3	32 (39.5%)	17 (38.6%)	41 (36.3%)	248 (57.9%)	338 (50.8%)
4	19 (23.5%)	7 (15.9%)	42 (37.2%)	83 (19.4%)	151 (22.7%)
5	24 (29.6%)	4 (9.1%)	30 (26.5%)	66 (15.4%)	124 (18.6%)

## Data Availability

All data can be made available upon request from the corresponding author.

## Supplementary Materials

The following supporting information can be downloaded at: https://www.mdpi.com/article/10.3390/vaccines14080688/s1, Figure S1: Anti-SARS-CoV-2 Spike IgG levels post-vaccination in serum samples from autoimmune cohorts; Figure S2: Anti-SARS-CoV-2 Spike IgG levels post-vaccination in serum samples from transplant cohorts stratified by transplant type; Figure S3: Anti-SARS-CoV-2 Spike IgG avidity post-vaccination in serum samples from autoimmune cohorts; Figure S4: Anti-SARS-CoV-2 Spike IgG avidity post-vaccination in serum samples from transplant cohorts stratified by transplant type; Figure S5: Anti-SARS-CoV-2 Spike IgG levels post-vaccination in serum samples from autoimmune disease, IBD, transplant, and healthy cohorts within different age groups (<65 years and ≥65 years); Figure S6: Anti-SARS-CoV-2 Spike IgG avidity post-vaccination in serum samples from autoimmune disease, IBD, transplant, and healthy cohorts within different age groups (<65 years and ≥65 years); Figure S7: Anti-SARS-CoV-2 Spike IgG levels post-vaccination in serum samples from autoimmune disease, IBD, transplant, and healthy cohorts within different sex groups; Figure S8: Anti-SARS-CoV-2 Spike IgG avidity post-vaccination in serum samples from autoimmune disease, IBD, transplant, and healthy cohorts within different sex groups; Figure S9: Anti-SARS-CoV-2 Spike IgG levels post-vaccination in serum samples from autoimmune disease, IBD, transplant, and healthy cohorts within different vaccine groups; Figure S10: Anti-SARS-CoV-2 Spike IgG avidity post-vaccination in serum samples from autoimmune disease, IBD, transplant, and healthy cohorts within different vaccine groups; Table S1: Treatment information of the study participants; Table S2: Anti-spike IgG levels in each cohort; Table S3: Systemic and non-systemic autoimmune conditions in participants; Table S4: Positive and negative percentage of anti-nucleocapsid IgG in each cohort; Table S5: Anti-spike IgG Avidity in each cohort.
