# Supplementary material for: Circulating SARS-CoV-2 Spike IgG Antibody Levels and Avidity in Autoimmune, IBD and Transplant Cohorts: An Observational Study Following Multiple COVID-19 Vaccine Boosters
Source: Vaccines (Basel). 2026 Aug 11;14(8):688. doi: 10.3390/vaccines14080688 (PMC13517781; doi:10.3390/vaccines14080688)
Supplement: Supplementary file 1 [file vaccines-14-00688-s001.zip › vaccines-4437105-supplementary.pdf]

# *Supplementary Material*

## 1 Supplementary Tables

### 1.1 Supplementary Table S1. Treatment information of the study participants

Supplementary Table S1a.

| Autoimmune Cohort | | | | |
|---|---|---|---|---|
| Treatment type | Dose | | | |
| | 2 | 3 | 4 | 5 |
| Corticosteroid | 0 | 0 | 0 | 1 |
| Conventional Synthetic DMARD | 1 | 1 | 1 | 0 |
| Combined | 0 | 0 | 0 | 0 |
| Unreported | 5 | 31 | 18 | 23 |

Supplementary Table S1b.

| IBD Cohort | | | | |
|---|---|---|---|---|
| Treatment type | Dose | | | |
| | 2 | 3 | 4 | 5 |
| Corticosteroid | 0 | 1 | 0 | 0 |
| Conventional Synthetic DMARD | 1 | 0 | 0 | 0 |
| Biologic DMARD | 5 | 6 | 0 | 0 |
| -- Anti-TNF | 1 | 4 | 0 | 0 |
| Targeted Synthetic DMARD | 1 | 0 | 0 | 0 |
| Immunomodulator | 1 | 0 | 0 | 0 |
| Combined | 2 | 4 | 2 | 0 |
| Non-immunosuppressive | 1 | 0 | 0 | 0 |
| Unreported | 4 | 2 | 5 | 4 |

Supplementary Table S1c.

| Transplant Cohort | | | |
|---|---|---|---|
| Treatment type | Dose | | |
| | 3 | 4 | 5 |
| Antimetabolite | 1 | 0 | 0 |
| Belatacept | 1 | 0 | 0 |
| Calcineurin inhibitor | 5 | 0 | 0 |

| | | | |
|---|---|---|---|
| Combined | 23 | 2 | 0 |
| Non-immunosuppressive | 5 | 0 | 0 |
| Unreported | 5 | 40 | 30 |

1.2 Supplementary Table S2. Anti-spike IgG levels in each cohort

| Cohort | | | N | Geometric mean concentration (BAU/mL) | 95% CI | p-value when compared to Healthy * | p-value when compared to Matched Healthy Control* |
|---|---|---|---|---|---|---|---|
| 1-month | 2 doses | Healthy | 30 | 2725 | 2077-3577 | NA | NA |
| | | Autoimmune | 5 | 3834 | 1777-8271 | 0.3447 | 0.2858 |
| | | IBD | 16 | 1628 | 818-3239 | 0.264 | 0.4523 |
| | | Transplant | 0 | NA | NA | NA | NA |
| | 3 doses | Healthy | 244 | 7685 | 7014-8420 | NA | NA |
| | | Autoimmune | 32 | 5563 | 3316-9331 | 0.7142 | 0.7002 |
| | | IBD | 16 | 5292 | 3576-7831 | 0.0779 | 0.3183 |
| | | Transplant | 38 | 635 | 271-1488 | <0.0001 | <0.001 |
| | 4 doses | Healthy | 82 | 8805 | 7325-10585 | NA | NA |
| | | Autoimmune | 17 | 5942 | 3734-9457 | 0.1365 | 0.1341 |
| | | IBD | 5 | 4200 | 2251-7837 | 0.1008 | 0.0477 |
| | | Transplant | 31 | 5020 | 2711-9293 | 0.1365 | 0.2116 |
| | 5 doses | Healthy | 66 | 10352 | 8517-12583 | NA | NA |
| | | Autoimmune | 23 | 9165 | 5958-14099 | 0.8771 | 1 |
| | | IBD | 4 | 5362 | 1477-19462 | 0.1796 | 0.1715 |
| | | Transplant | 30 | 6365 | 4132-9805 | 0.1485 | 0.1715 |
| 6-month | 2 doses | Healthy | 30 | 864 | 501-1491 | NA | NA |
| | | Autoimmune | 6 | 1457 | 540-3932 | 0.2683 | 0.9048 |
| | | IBD | 13 | 433 | 156-1202 | 0.2683 | 0.9048 |
| | | Transplant | 0 | NA | NA | NA | NA |
| | 3 doses | Healthy | 240 | 3277 | 2916-3683 | NA | NA |
| | | Autoimmune | 32 | 1911 | 1143-3198 | 0.0576 | 0.0992 |
| | | IBD | 17 | 2102 | 1251-3530 | 0.1184 | 0.2255 |
| | | Transplant | 40 | 1146 | 608-2160 | 0.0057 | 0.0992 |
| | 4 doses | Healthy | 81 | 5385 | 4583-6328 | NA | NA |
| | | Autoimmune | 18 | 2893 | 1689-4956 | 0.056 | 0.3095 |
| | | IBD | 6 | 1689 | 419-6808 | 0.056 | 0.3095 |
| | | Transplant | 42 | 3743 | 2617-5353 | 0.1583 | 0.5796 |
| | 5 doses | Healthy | 65 | 5311 | 4159-6781 | NA | NA |
| | | Autoimmune | 24 | 4840 | 2865-8178 | 0.9594 | 0.4041 |
| | | IBD | 4 | 2184 | 278-17142 | 0.2312 | 0.4041 |

| | | | | | | | |
|---|---|---|---|---|---|---|---|
| | | Transplant | 29 | 2897 | 1647-5096 | 0.1107 | 0.162 |

*Differences were assessed using the nonparametric Wilcoxon rank-sum test. P values were adjusted using the Benjamini-Hochberg FDR method within each vaccine dose and post-vaccination interval.

## 1.3 Supplementary Table S3. Systemic and non-systemic autoimmune conditions in participants

| Systemic | Non-systemic |
| --- | --- |
| • Rheumatoid Arthritis<br>• Sjögren Syndrome<br>• Systemic Lupus Erythematosus | • Alopecia areata<br>• Arthropathic psoriasis<br>• Atopic dermatitis<br>• Autoimmune disorder, type unspecified<br>• Autoimmune encephalitis<br>• Celiac disease<br>• Cicatricial alopecia<br>• Fibromyalgia<br>• Giant cell arteritis<br>• Hashimoto's Disease<br>• Immune thrombocytopenic purpura<br>• Multiple sclerosis<br>• Myositis<br>• Primary biliary cirrhosis<br>• Raynaud's syndrome<br>• Type 1 diabetes mellitus without complications<br>• Unspecified contact dermatitis<br>• Vitiligo |

## 1.4 Supplementary Table S4. Positive and negative percentage of anti-nucleocapsid IgG in each cohort

| Cohort | | | N | Negative | Positive | p-value* |
|---|---|---|---|---|---|---|
| 1-month | 2 doses | Healthy | 30 | 30 (100.0%) | 0 (0.0%) | -- |
| | | Autoimmune | 5 | 4 (80.0%) | 1 (20.0%) | 0.6008 |
| | | IBD | 16 | 16 (100.0%) | 0 (0.0%) | 1 |
| | | Transplant | 0 | -- | -- | -- |
| | 3 doses | Healthy | 244 | 216 (88.5%) | 28 (11.5%) | -- |
| | | Autoimmune | 32 | 32 (100.0%) | 0 (0.0%) | 0.2502 |
| | | IBD | 16 | 15 (93.8%) | 1 (6.2%) | 0.8155 |
| | | Transplant | 38 | 37 (97.4%) | 1 (2.6%) | 0.2502 |
| | 4 doses | Healthy | 82 | 68 (82.9%) | 14 (17.1%) | -- |
| | | Autoimmune | 17 | 16 (94.1%) | 1 (5.9%) | 0.6360 |
| | | IBD | 5 | 5 (100.0%) | 0 (0.0%) | 0.7026 |
| | | Transplant | 31 | 29 (93.5%) | 2 (6.5%) | 0.6360 |
| | 5 doses | Healthy | 66 | 54 (81.8%) | 12 (18.2%) | -- |
| | | Autoimmune | 23 | 22 (100.0%) | 0 (0.0%) | 0.2187 |
| | | IBD | 4 | 3 (75.0%) | 1 (25.0%) | 1 |
| | | Transplant | 30 | 27 (93.1%) | 2 (6.9%) | 0.3975 |
| 6-month | 2 doses | Healthy | 30 | 24 (80.0%) | 6 (20.0%) | -- |
| | | Autoimmune | 6 | 4 (66.7%) | 2 (33.3%) | 0.8577 |
| | | IBD | 13 | 12 (92.3%) | 1 (7.7%) | 0.8577 |
| | | Transplant | 0 | -- | -- | -- |
| | 3 doses | Healthy | 240 | 189 (78.8%) | 51 (21.2%) | -- |
| | | Autoimmune | 32 | 30 (93.8%) | 2 (6.2%) | 0.1139 |
| | | IBD | 17 | 13 (76.5%) | 4 (23.5%) | 1 |
| | | Transplant | 40 | 37 (92.5%) | 3 (7.5%) | 0.1139 |
| | 4 doses | Healthy | 81 | 66 (81.5%) | 15 (18.5%) | -- |
| | | Autoimmune | 18 | 16 (88.9%) | 2 (11.1%) | 1 |
| | | IBD | 6 | 5 (83.3%) | 1 (16.7%) | 1 |
| | | Transplant | 42 | 37 (90.2%) | 4 (9.8%) | 0.9570 |
| | 5 doses | Healthy | 65 | 54 (83.1%) | 11 (16.9%) | -- |
| | | Autoimmune | 24 | 19 (82.6%) | 4 (17.4%) | 1 |
| | | IBD | 4 | 2 (50.0%) | 2 (50.0%) | 0.9765 |
| | | Transplant | 29 | 25 (86.2%) | 4 (13.8%) | 1 |

*Differences were assessed using Pearson's chi-squared test. P values were adjusted using the Benjamini-Hochberg FDR method within each vaccine dose and post-vaccination interval.

## 1.5 Supplementary Table S5. Anti-spike IgG Avidity in each cohort

| Cohort | | | N | Geometric mean AI80* (M) | 95% CI | p-value when compared to Healthy ** | p-value when compared to Matched Healthy Control** |
|---|---|---|---|---|---|---|---|
| 1-month | 2 doses | Healthy | 31 | 2.9 | 2.6-3.2 | NA | NA |
| | | Autoimmune | 5 | 3.6 | 2.3-5.7 | 0.449 | 0.7857 |
| | | IBD | 16 | 2.8 | 2.4-3.3 | 0.714 | 0.2112 |
| | | Transplant | 0 | NA | NA | NA | NA |
| | 3 doses | Healthy | 246 | 5.5 | 5.4-5.6 | NA | NA |
| | | Autoimmune | 32 | 5 | 4.7-5.3 | 0.0084 | 0.5663 |
| | | IBD | 16 | 5 | 4.5-5.5 | 0.0704 | 0.5872 |
| | | Transplant | 28 | 4.5 | 4.2-4.8 | <0.0001 | <0.001 |
| | 4 doses | Healthy | 83 | 5.8 | 5.6-5.9 | NA | NA |
| | | Autoimmune | 17 | 5.3 | 4.9-5.8 | 0.0707 | 0.1136 |
| | | IBD | 5 | 5.2 | 4.4-6.1 | 0.0707 | 0.0477 |
| | | Transplant | 29 | 4.9 | 4.5-5.2 | <0.0001 | 0.018 |
| | 5 doses | Healthy | 68 | 5.7 | 5.5-5.9 | NA | NA |
| | | Autoimmune | 23 | 5.4 | 5.2-5.7 | 0.1091 | 0.2954 |
| | | IBD | 4 | 5.5 | 4.9-6.1 | 0.2905 | 1 |
| | | Transplant | 30 | 5.1 | 4.7-5.5 | 0.0156 | 0.0576 |
| 6-month | 2 doses | Healthy | 32 | 4.1 | 3.7-4.6 | NA | NA |
| | | Autoimmune | 6 | 4.6 | 3.9-5.3 | 0.6818 | 0.381 |
| | | IBD | 13 | 3.1 | 2.5-3.9 | 0.0356 | 0.2292 |
| | | Transplant | 0 | NA | NA | NA | NA |
| | 3 doses | Healthy | 242 | 5.4 | 5.3-5.5 | NA | NA |
| | | Autoimmune | 31 | 4.8 | 4.4-5.1 | 0.0017 | 0.0683 |
| | | IBD | 17 | 4.6 | 4.1-5.1 | 0.0017 | 0.0683 |
| | | Transplant | 35 | 4.6 | 4.2-5 | <0.0001 | 0.0369 |
| | 4 doses | Healthy | 82 | 5.9 | 5.7-6 | NA | NA |
| | | Autoimmune | 18 | 5.1 | 4.3-6 | 0.0354 | 0.3189 |
| | | IBD | 6 | 5.1 | 4.5-5.8 | 0.0182 | 0.0143 |
| | | Transplant | 41 | 4.5 | 4.2-4.8 | <0.0001 | <0.0001 |
| | 5 doses | Healthy | 66 | 5.8 | 5.6-6 | NA | NA |
| | | Autoimmune | 24 | 5.4 | 5.1-5.7 | 0.0381 | 0.278 |
| | | IBD | 4 | 5.8 | 4.4-7.6 | 0.81 | 1 |

| | | | | | | | |
|---|---|---|---|---|---|---|---|
| | | Transplant | 28 | 4.7 | 4.3-5.2 | <0.0001 | 0.0045 |

*Avidity is reported as AI80, defined as the molar concentration of urea (M) required to reduce the optical density signal to 80% of the corresponding untreated wells.

**Differences were assessed using the nonparametric Wilcoxon rank-sum test. P values were adjusted using the Benjamini-Hochberg FDR method within each vaccine dose and post-vaccination interval.

## 2 Supplementary Figures

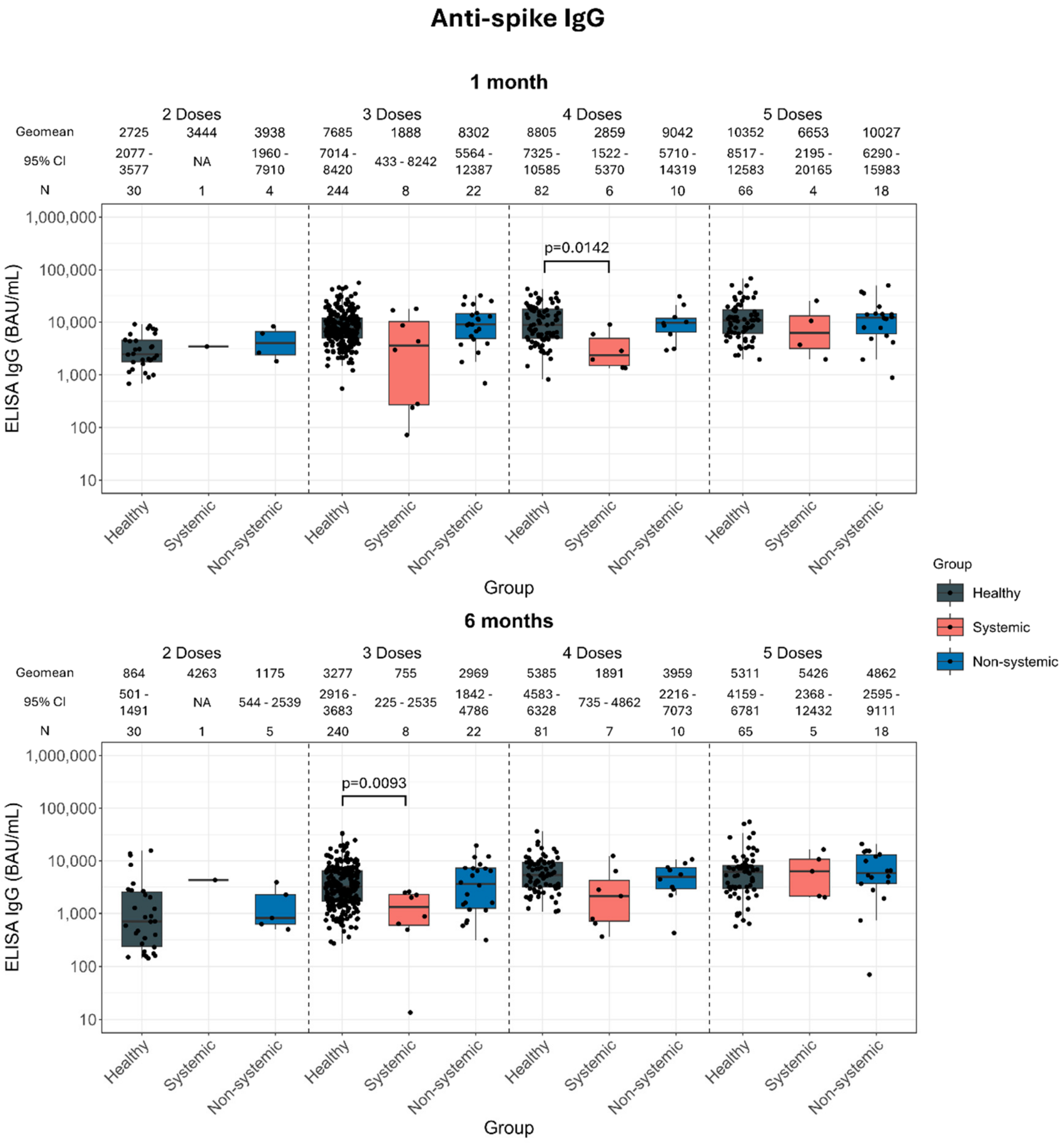


**Supplementary Figure S1. Anti-SARS-CoV-2 Spike IgG levels post-vaccination in serum samples from autoimmune cohorts.**

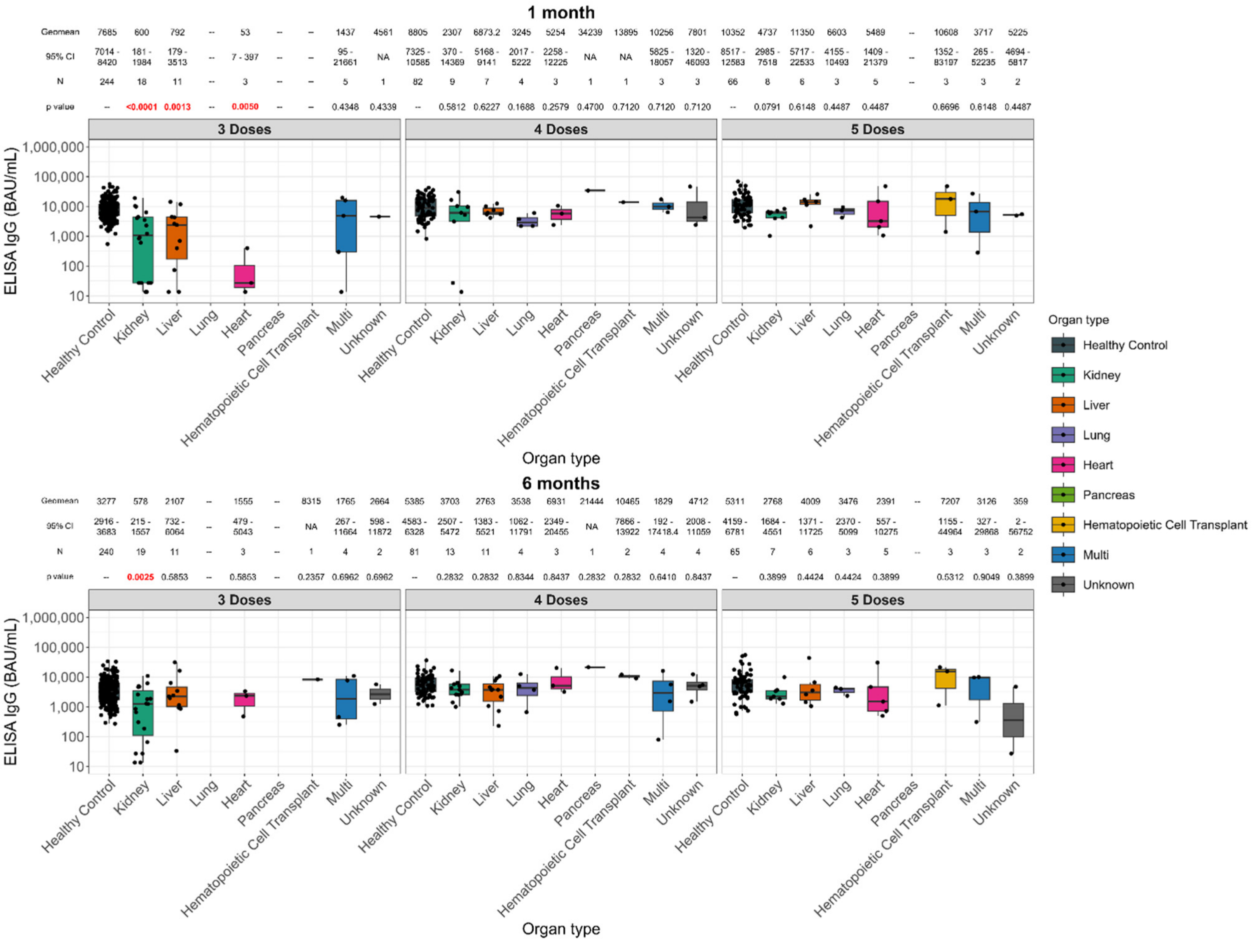


**Supplementary Figure S2. Anti-SARS-CoV-2 Spike IgG levels post-vaccination in serum samples from transplant cohorts stratified by transplant type.**

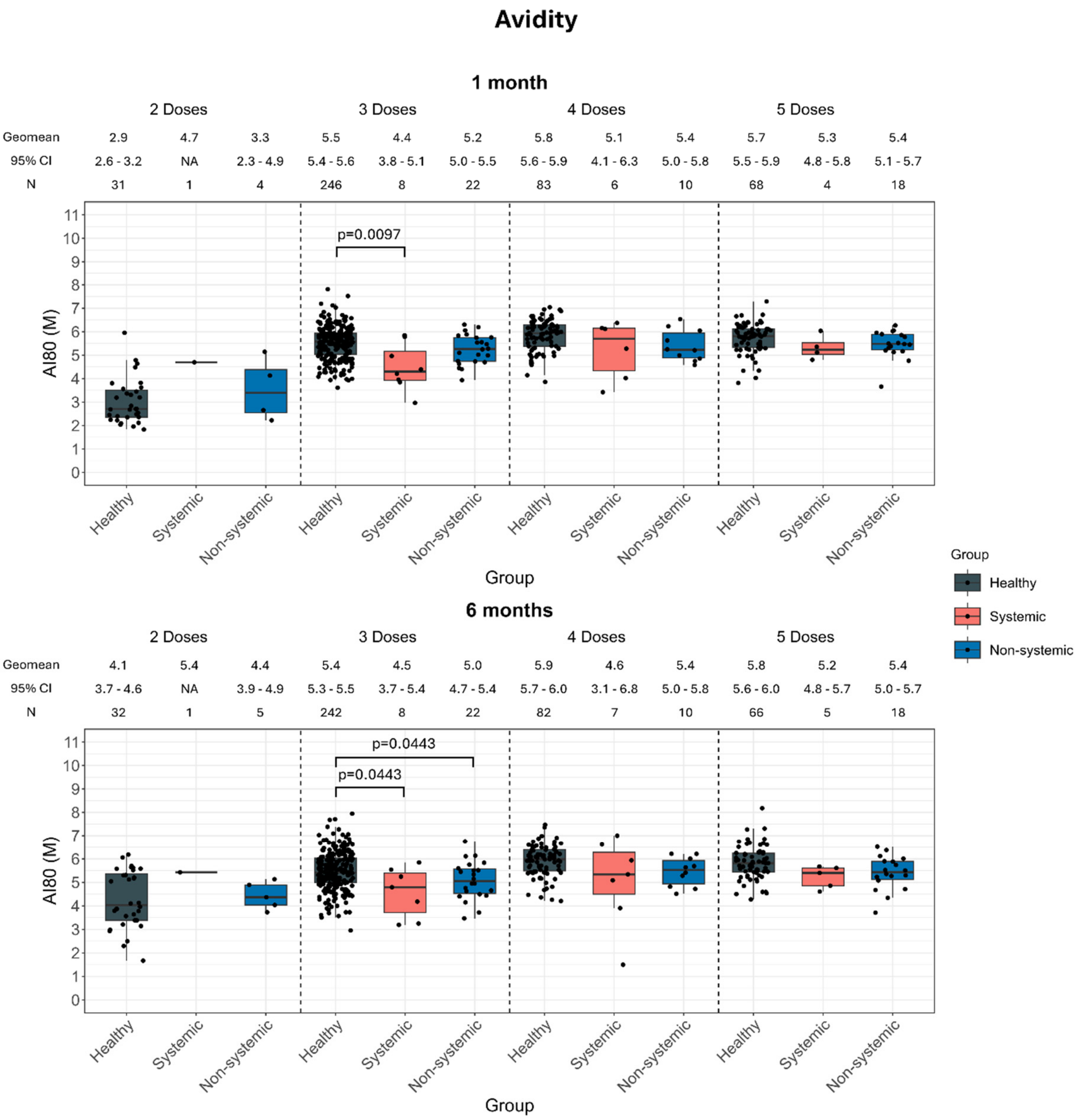


**Supplementary Figure S3. Anti-SARS-CoV-2 Spike IgG avidity post-vaccination in serum samples from autoimmune cohorts.**

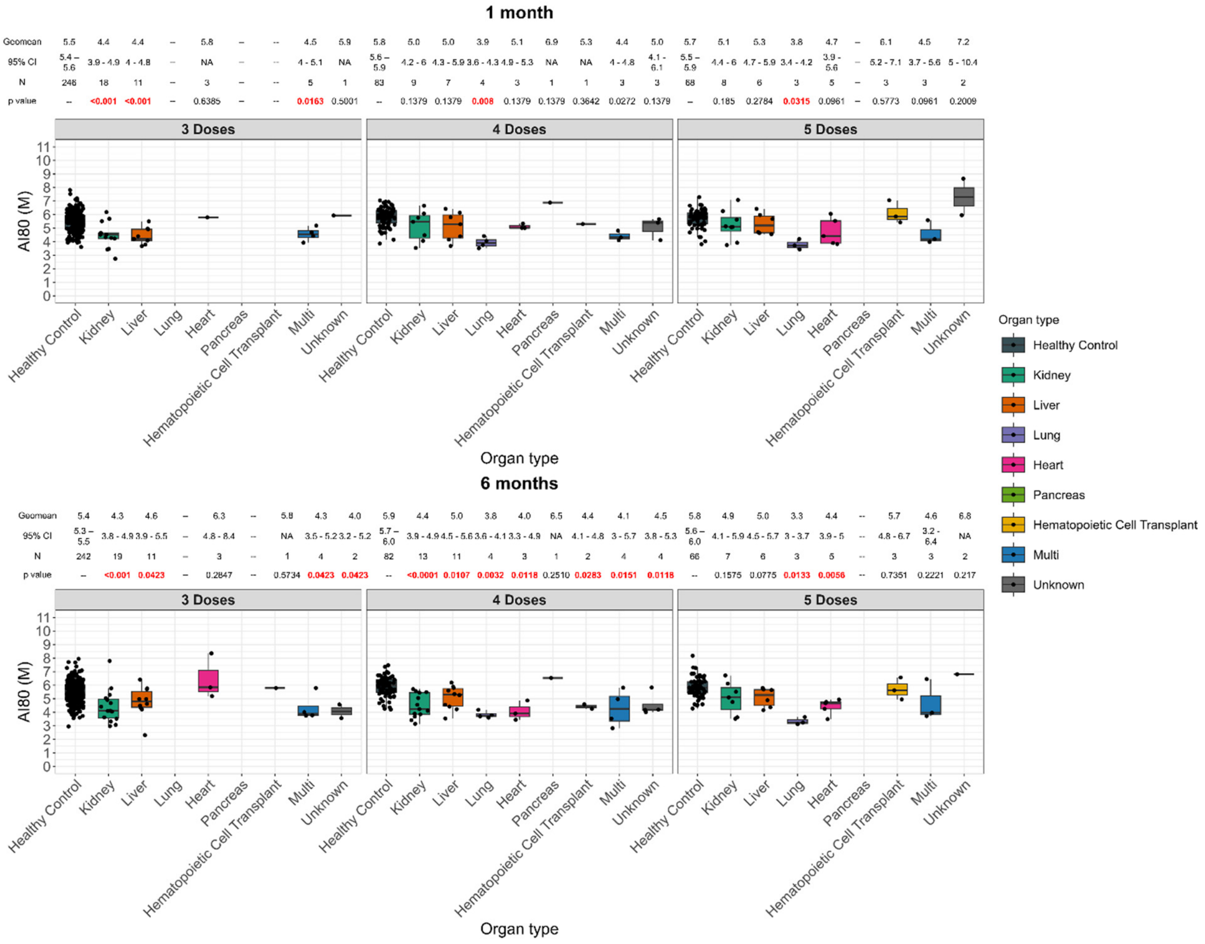


**Supplementary Figure S4. Anti-SARS-CoV-2 Spike IgG avidity post-vaccination in serum samples from transplant cohorts stratified by transplant type.**

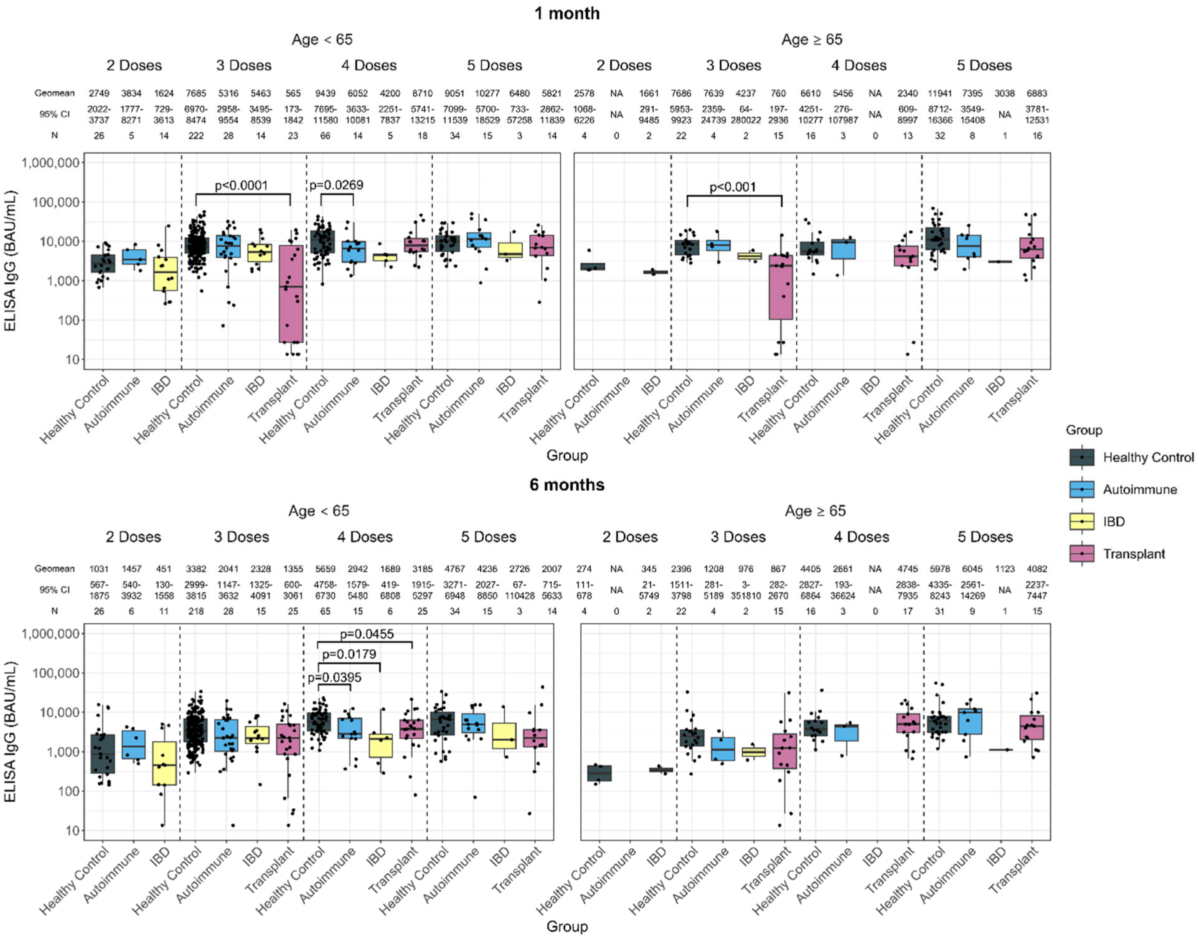


**Supplementary Figure S5. Anti-SARS-CoV-2 Spike IgG levels post-vaccination in serum samples from autoimmune disease, IBD, transplant, and healthy cohorts within different age groups (<65 years and ≥ 65 years).**

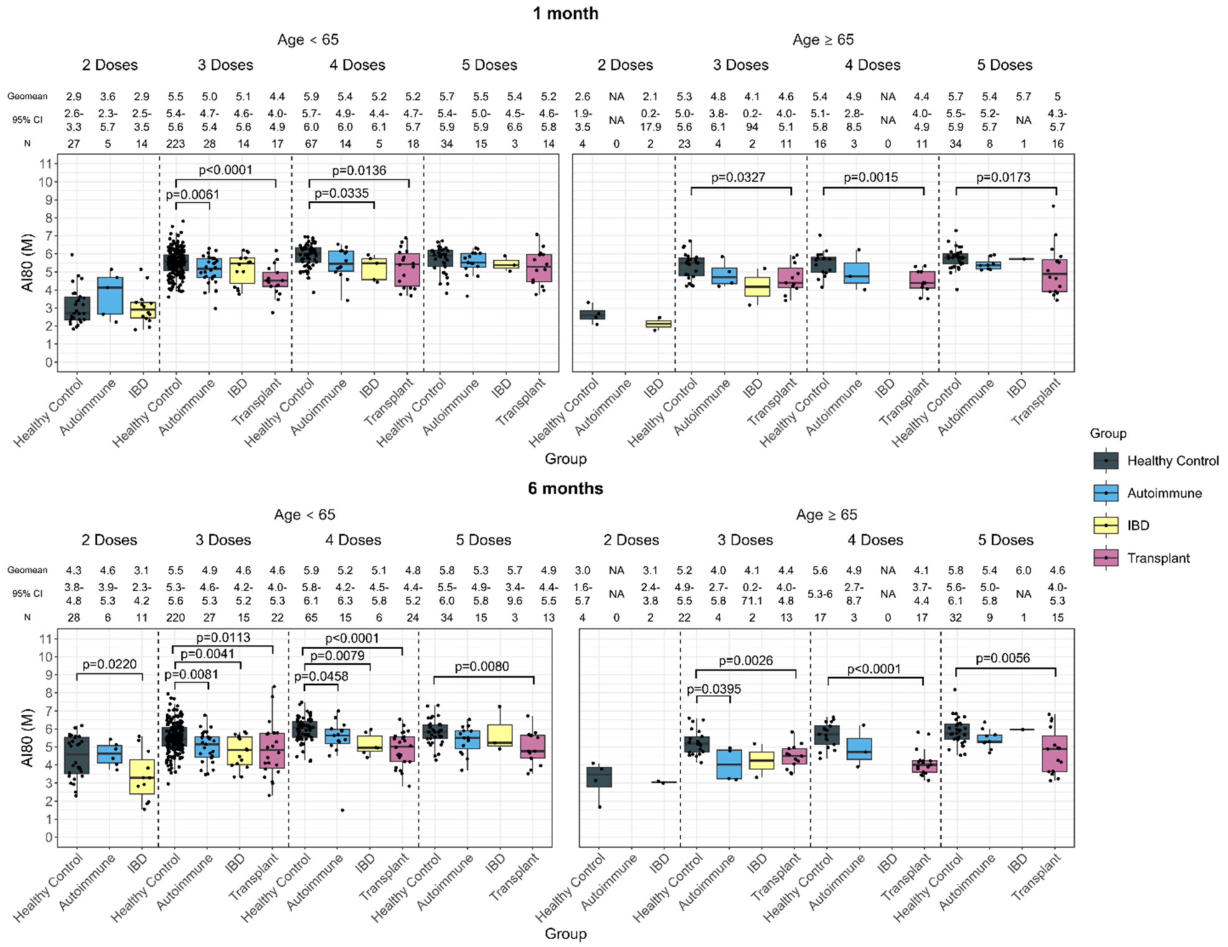


**Supplementary Figure S6. Anti-SARS-CoV-2 Spike IgG avidity post-vaccination in serum samples from autoimmune disease, IBD, transplant, and healthy cohorts within different age groups (<65 years and ≥ 65 years).**

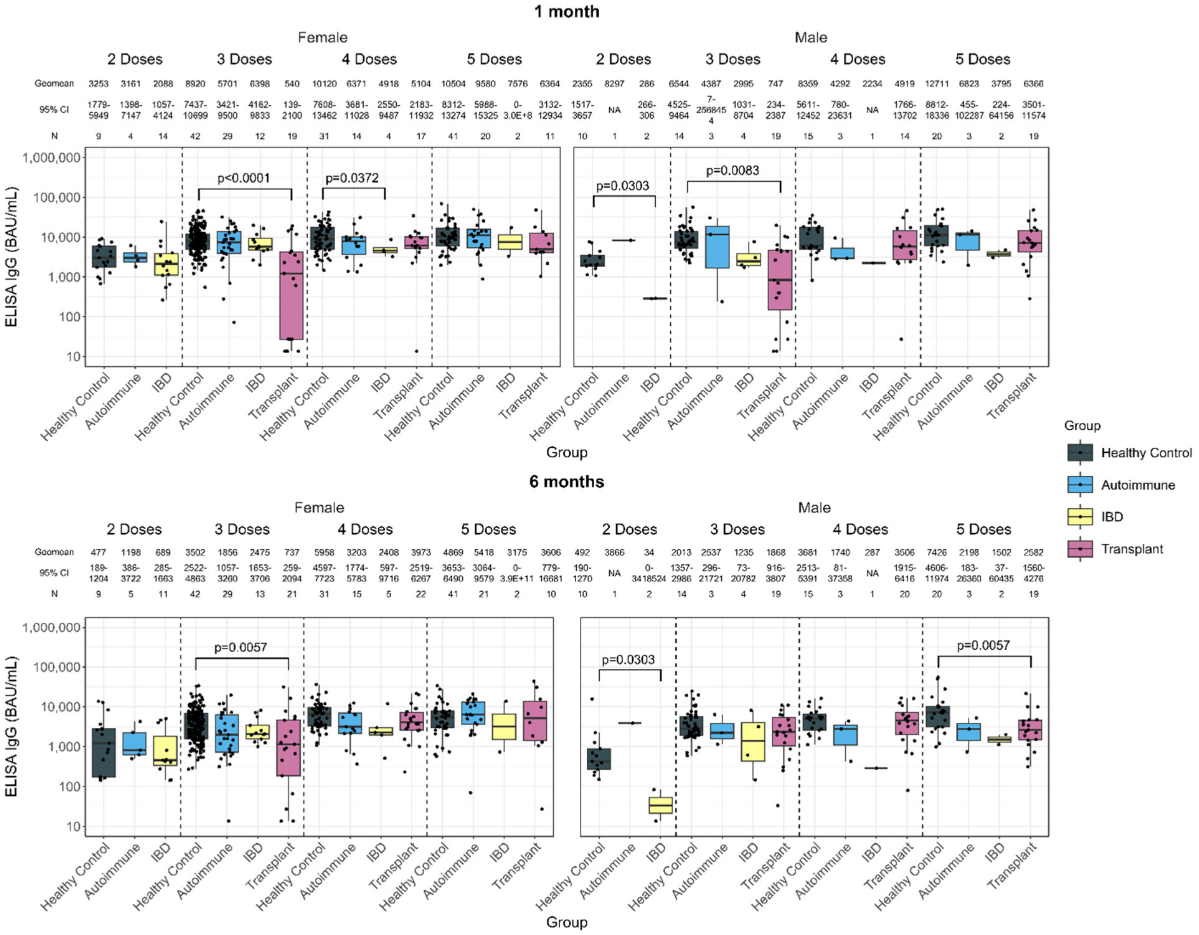


**Supplementary Figure S7. Anti-SARS-CoV-2 Spike IgG levels post-vaccination in serum samples from autoimmune disease, IBD, transplant, and healthy cohorts within different sex groups.**

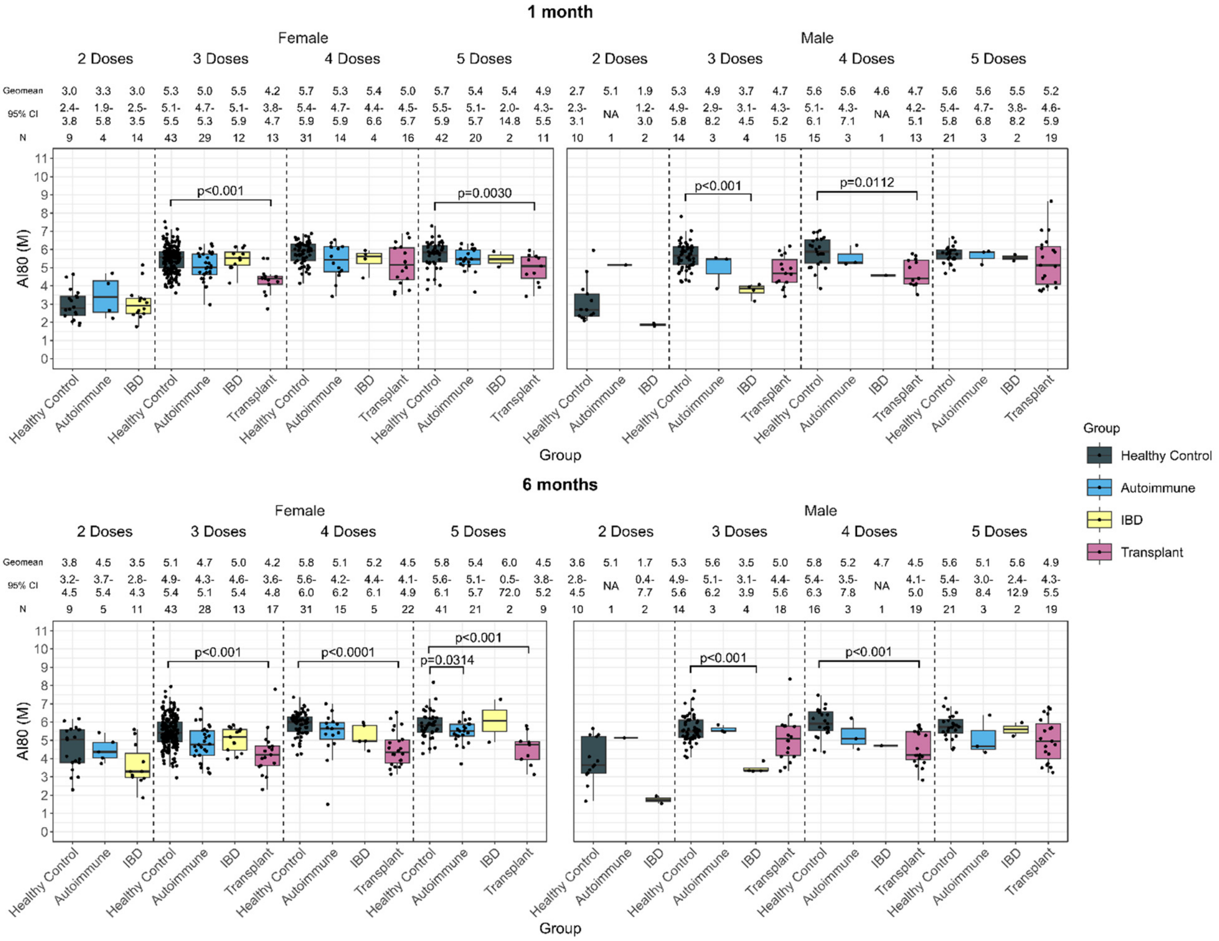


**Supplementary Figure S8. Anti-SARS-CoV-2 Spike IgG avidity post-vaccination in serum samples from autoimmune disease, IBD, transplant, and healthy cohorts within different sex groups.**

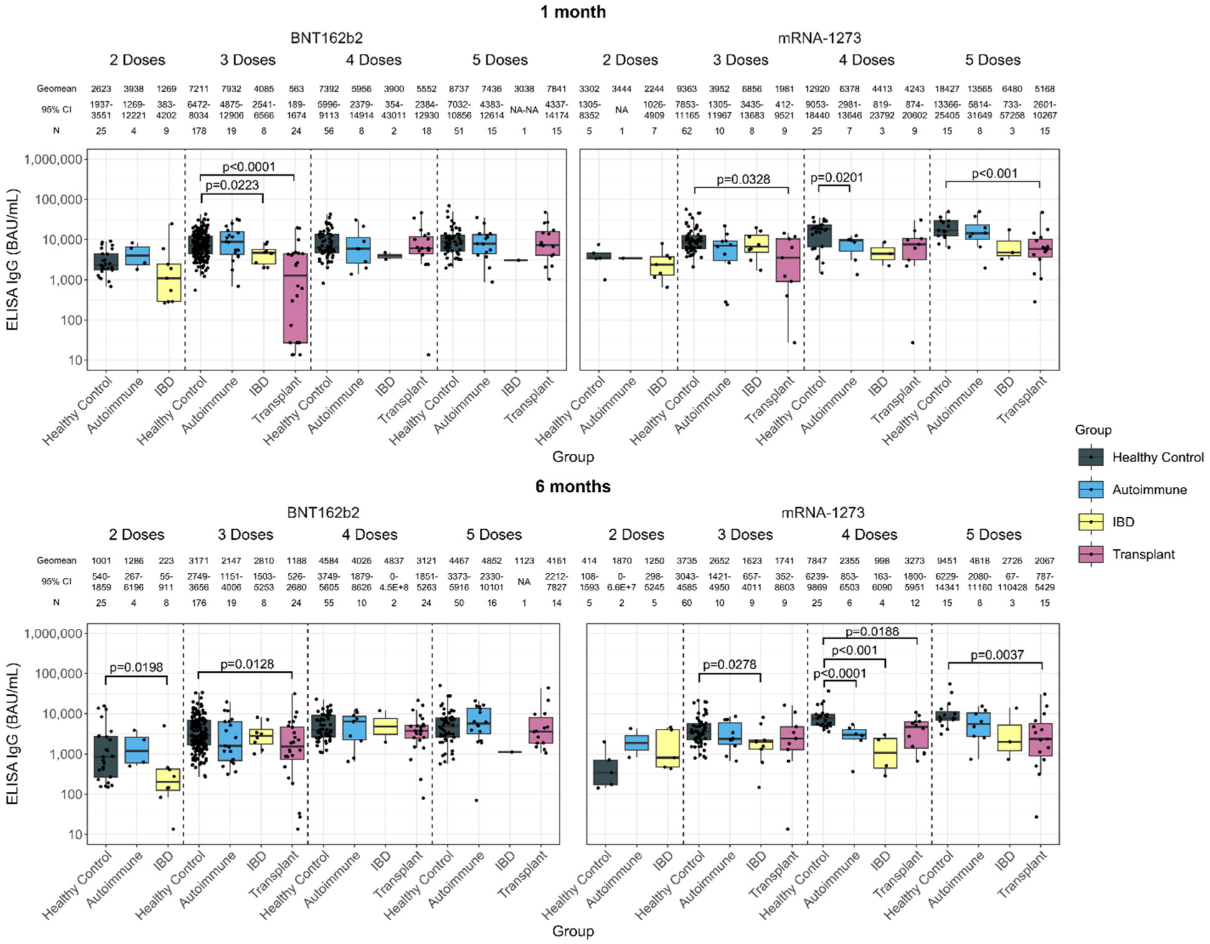


**Supplementary Figure S9. Anti-SARS-CoV-2 Spike IgG levels post-vaccination in serum samples from autoimmune disease, IBD, transplant, and healthy cohorts within different vaccine groups.**

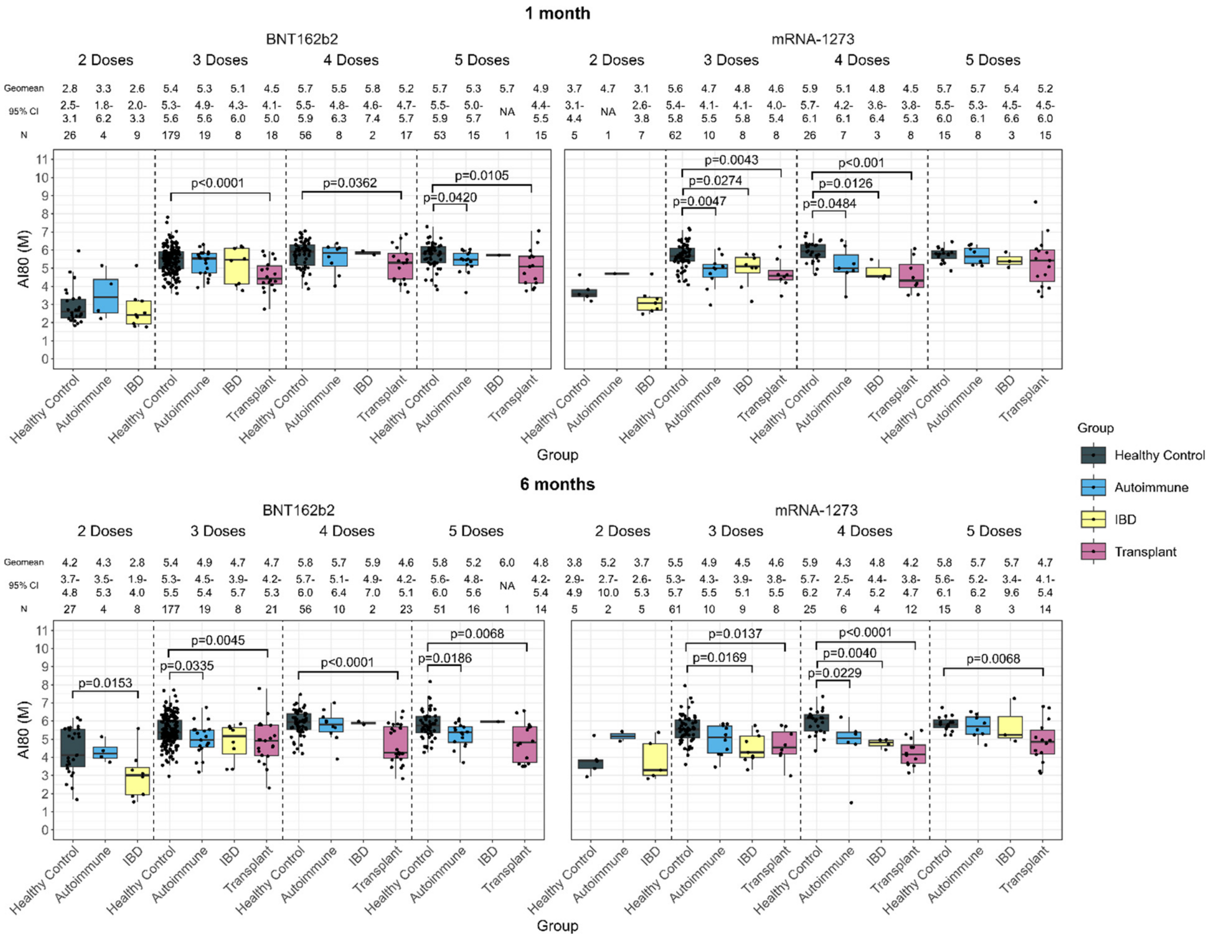


**Supplementary Figure S10. Anti-SARS-CoV-2 Spike IgG avidity post-vaccination in serum samples from autoimmune disease, IBD, transplant, and healthy cohorts within different vaccine groups.**

## 3 Supplementary Figures Caption

**Supplementary Figure S1. Anti-SARS-CoV-2 Spike IgG levels post-vaccination in serum samples from autoimmune cohorts.** Comparison of serum anti-spike IgG levels in the healthy, systemic autoimmune and non-systemic autoimmune cohorts at 1-month and 6-month post-vaccination. Anti-spike IgG levels were presented as Geometric Mean (GM) with 95% confidence intervals (95% CI). Box plots show median (horizontal bar), the first and third quartiles. Differences assessed by non-parametric Wilcoxon rank sum test. P values were adjusted using the Benjamini-Hochberg false discovery rate method within each dose and post-vaccination interval. Adjusted p values $< 0.05$ were considered statistically significant.

**Supplementary Figure S2. Anti-SARS-CoV-2 Spike IgG levels post-vaccination in serum samples from transplant cohorts stratified by transplant type.** Comparison of serum anti-spike IgG levels in the healthy cohort and transplant cohorts stratified by transplant type at 1-month and 6-month post-vaccination. Anti-spike IgG levels were presented as Geometric Mean (GM) with 95% confidence intervals (95% CI). Box plots show median (horizontal bar), the first and third quartiles. Differences assessed by non-parametric Wilcoxon rank sum test. P values were adjusted using the Benjamini-Hochberg false discovery rate method within each dose and post-vaccination interval. Adjusted p values $< 0.05$ were considered statistically significant.

**Supplementary Figure S3. Anti-SARS-CoV-2 Spike IgG avidity post-vaccination in serum samples from autoimmune cohorts.** Comparison of serum anti-spike IgG avidity in the healthy, systemic autoimmune and non-systemic autoimmune cohorts at 1-month and 6-month post-vaccination. Anti-spike IgG avidity was presented as Geometric Mean (GM) with 95% confidence intervals (95% CI). Box plots show median (horizontal bar), the first and third quartiles. Differences assessed by non-parametric Wilcoxon rank sum test. P values were adjusted using the Benjamini-Hochberg false discovery rate method within each dose and post-vaccination interval. Adjusted p values $< 0.05$ were considered statistically significant.

**Supplementary Figure S4. Anti-SARS-CoV-2 Spike IgG avidity post-vaccination in serum samples from transplant cohorts stratified by transplant type.** Comparison of serum anti-spike IgG avidity in the healthy cohort and transplant cohorts stratified by transplant type at 1-month and 6-month post-vaccination. Anti-spike

IgG avidity was presented as Geometric Mean (GM) with 95% confidence intervals (95% CI). Box plots show median (horizontal bar), the first and third quartiles. Differences assessed by non-parametric Wilcoxon rank sum test. P values were adjusted using the Benjamini-Hochberg false discovery rate method within each dose and post-vaccination interval. Adjusted p values < 0.05 were considered statistically significant.

**Supplementary Figure S5. Anti-SARS-CoV-2 Spike IgG levels post-vaccination in serum samples from autoimmune disease, IBD, transplant, and healthy cohorts within different age groups (<65 years and ≥ 65 years).** Comparison of serum anti-spike IgG levels in healthy, autoimmune, IBD and transplant cohorts within different age groups 1-month and 6-month post-vaccination. Anti-spike IgG levels were presented as Geometric Mean (GM) with 95% confidence intervals (95% CI). Box plots show median (horizontal bar), the first and third quartiles. Differences assessed by non-parametric Wilcoxon rank sum test.

**Supplementary Figure S6. Anti-SARS-CoV-2 Spike IgG avidity post-vaccination in serum samples from autoimmune disease, IBD, transplant, and healthy cohorts within different age groups (<65 years and ≥ 65 years).** Comparison of serum anti-spike IgG avidity in healthy, autoimmune, IBD and transplant cohorts within different age groups 1-month and 6-month post-vaccination. Anti-spike IgG avidity was presented as Geometric Mean (GM) with 95% confidence intervals (95% CI). Box plots show median (horizontal bar), the first and third quartiles. Differences assessed by non-parametric Wilcoxon rank sum test.

**Supplementary Figure S7. Anti-SARS-CoV-2 Spike IgG levels post-vaccination in serum samples from autoimmune disease, IBD, transplant, and healthy cohorts within different sex groups.** Comparison of serum anti-spike IgG levels in healthy, autoimmune, IBD and transplant cohorts within different sex groups 1-month and 6-month post-vaccination. Anti-spike IgG levels were presented as Geometric Mean (GM) with 95% confidence intervals (95% CI). Box plots show median (horizontal bar), the first and third quartiles. Differences assessed by non-parametric Wilcoxon rank sum test.

**Supplementary Figure S8. Anti-SARS-CoV-2 Spike IgG avidity post-vaccination in serum samples from autoimmune disease, IBD, transplant, and healthy cohorts within different sex groups.** Comparison of

serum anti-spike IgG avidity in healthy, autoimmune, IBD and transplant cohorts within different sex groups 1-month and 6-month post-vaccination. Anti-spike IgG avidity was presented as Geometric Mean (GM) with 95% confidence intervals (95% CI). Box plots show median (horizontal bar), the first and third quartiles. Differences assessed by non-parametric Wilcoxon rank sum test.

**Supplementary Figure S9. Anti-SARS-CoV-2 Spike IgG levels post-vaccination in serum samples from autoimmune disease, IBD, transplant, and healthy cohorts within different vaccine groups.** Comparison of serum anti-spike IgG levels in healthy, autoimmune, IBD and transplant cohorts within different vaccine groups 1-month and 6-month post-vaccination. Anti-spike IgG levels were presented as Geometric Mean with 95% confidence intervals (95% CI). Box plots show median (horizontal bar), the first and third quartiles. Differences assessed by non-parametric Wilcoxon rank sum test.

**Supplementary Figure S10. Anti-SARS-CoV-2 Spike IgG avidity post-vaccination in serum samples from autoimmune disease, IBD, transplant, and healthy cohorts within different vaccine groups.** Comparison of serum anti-spike IgG avidity in healthy, autoimmune, IBD and transplant cohorts within different vaccine groups 1-month and 6-month post-vaccination. Anti-spike IgG levels were presented as Geometric Mean with 95% confidence intervals (95% CI). Box plots show median (horizontal bar), the first and third quartiles. Differences assessed by non-parametric Wilcoxon rank sum test.
